# Impact of Tungsten Loading on the Activation of Zeolite-Based Catalysts for Methane Dehydroaromatization

**DOI:** 10.1021/acscatal.4c07228

**Published:** 2025-04-18

**Authors:** Josepha J.G. Kromwijk, Job G.A. Vloedgraven, Fleur Neijenhuis, Ward van der Stam, Matteo Monai, Bert M. Weckhuysen

**Affiliations:** Inorganic Chemistry and Catalysis Group, Institute for Sustainable and Circular Chemistry, Department of Chemistry, Utrecht University, Universiteitsweg 99, 3584 CG Utrecht, Netherlands

**Keywords:** methane dehydroaromatization, W/ZSM-5, tungsten, zeolite, operando spectroscopy, catalyst activation

## Abstract

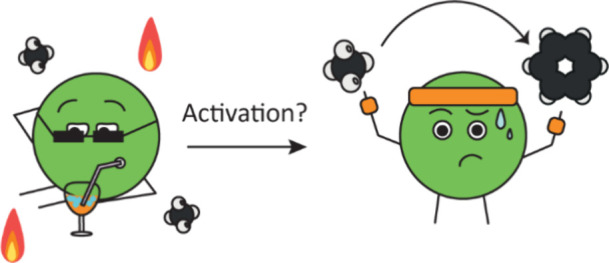

To improve the performance of zeolite-based catalysts for the methane dehydroaromatization (MDA) reaction, it is of importance to understand the nature of the catalytically active phase. Although many studies have been devoted to unraveling the structure of the active site, there is still no consensus. Monomeric, dimeric, and/or clusters of molybdenum oxide or tungsten oxide are proposed precatalyst structures. This precatalyst is activated under reaction conditions, to form (oxy)carbidic species which are believed to be the active site. In this study, we investigated the effect of tungsten dispersion on the activation of W/ZSM-5 catalysts. We observed unexpected long activation times that could be shortened by inert or reductive pretreatment. Based on our investigations, we hypothesize that W/ZSM-5 catalysts with low weight loadings (i.e., 2 wt %) cannot be activated due to the presence of monomeric tungsten. For catalysts with medium weight loadings (i.e., 5 and 7 wt %), restructuring of the tungsten is required for the formation of the active site, which can be achieved through performing a thermal pretreatment. For higher weight loadings (i.e., 10 wt %), reduction plays a key role in the activation of the catalyst. We show that the activation of the catalyst is impacted by the precatalyst structure. These insights aid in the development of suitable activation treatments which could save time and energy if the reaction would be performed at an industrial scale.

## Introduction

Annually, 139 billion cubic meters of methane are flared off as a byproduct of crude oil facilities worldwide.^1^ To mitigate this issue, crude oil facilities could implement on-site methane utilization. Considerable progress has already been made in the development of methane utilization technologies,^2^ and a reaction that has gained interest is methane dehydroaromatization (MDA). MDA is a highly endothermic reaction where methane is directly converted into aromatics at temperatures between 650 and 800 °C and under atmospheric pressure. The aromatic products formed during this process are valuable building blocks used for the production of plastics, medicine, and fuels. The demand for aromatics is growing, but the current production routes are still fossil fuel-based, so the MDA reaction is an interesting alternative.^3^ Mo/ZSM-5 is currently the most investigated catalyst material for the MDA reaction due to its outstanding performance compared to other metal-modified zeolites.^4,5^

Regardless of which metal-modified zeolite is used, the catalyst deactivates quickly under MDA reaction conditions because coke formation is thermodynamically favorable. Although the Mo/ZSM-5 catalyst is the most active, molybdenum brings an additional challenge to the table. Due to the volatile nature of MoO_3_, Mo migration on the zeolite support typically occurs.^6^ This way, MoC_*x*_ clusters are easily formed on the outer surface of the zeolite under reaction conditions, which are more prone to coking.^7^ To extend the catalyst lifetime reaction-regeneration cycles can be implemented,^8−11^ but during this process, aluminum molybdates are formed, which are inactive in the MDA reaction.^12^ For future industrial applications of the MDA process, it is of great interest to investigate alternative materials that are more resistant to the harsh MDA conditions, to ensure stable production of valuable hydrocarbons from methane.

Earlier studies show that several transition metals, like Fe, W, Cr, and V, can also be used as an active phase for MDA catalyst formulations.^5,13,14^ Of these alternative elements, tungsten shares similar chemical properties with molybdenum, but it has the advantage that it exhibits higher thermal stability. The Tamman temperature (i.e., the temperature at which metal oxides become mobile and are more prone to agglomeration) of WO_3_ compared MoO_3_ is significantly higher (600 °C compared to 261 °C).^15^ Furthermore, the formation of inactive aluminum tungstates has to our knowledge never been reported.^16^ This makes W/ZSM-5 an interesting alternative to the Mo/ZSM-5 catalyst for the MDA reaction. Nevertheless, the understanding of the structure-performance relationships of W/ZSM-5 remains incomplete. To understand the nature of the active phase of the W/ZSM-5 catalyst material, it is of essence to know how the precatalyst structure influences the activation, which will be the focus of our studies.

While the tungsten system for the MDA process is less studied, W/SiO_2_ catalysts have been explored extensively in the context of e.g., olefin metathesis.^17−19^ For this process, tungsten is the metal of choice due to its higher stability and resistance to poisoning compared to, for example, Mo/SiO_2_ and ReO_*x*_/Al_2_O_3_.^20−22^ During olefin metathesis, the activity typically increases slowly until a steady state is reached. In literature, this phenomenon is referred to as the “break-in” period.^23,24^ Howell et al. show that the duration of this period can be adjusted by pretreating the W/SiO_2_ catalyst in He instead of air. Raman spectroscopy and W L_1_-edge X-ray absorption near-edge spectroscopy (XANES) revealed that the sample pretreated in He resulted in a higher proportion of dioxo to mono-oxo tungsten species. These species would play a key role in forming the active site for olefin metathesis.^25^

Earlier studies show that the activation of W for MDA is quite demanding, which results in long activation times: similar to olefin metathesis there is a “break-in period.” Iglesia et al. showed that for the W/ZSM-5 catalyst, this induction period could take 2–4 h when the MDA reaction was performed at 700 °C,^26^ which is in stark contrast with the Mo/ZSM-5 catalyst, where typical induction periods of 20 min or less are observed. In their study, Iglesia et al. showed that the fresh catalyst consisted of isolated (WO_2_)^2+^ centers, which are converted to WC_*x*_ clusters of ∼0.6 nm during the activation period. However, in other studies, the formation of WC_*x*_ clusters was not observed. In an X-ray photoelectron spectroscopy (XPS) study, tungsten suboxides were observed after the catalyst was exposed to CH_4_,^27^ and also with ^13^C magic angle spinning (MAS) nuclear magnetic resonance (NMR), the presence of WC_*x*_ could not be confirmed.^28^ Therefore, there is no consensus on the active phase of the W/ZSM-5 catalyst.

In this work, we have investigated the activation mechanism of W/ZSM-5 catalysts for the methane dehydroaromatization (MDA) reaction as a function of W loading. The combination of ex situ characterization techniques, such as X-ray diffraction (XRD), NH_3_ temperature-programmed desorption (NH_3_-TPD), and Raman spectroscopy, with operando UV–vis spectroscopy and in situ X-ray diffraction (XRD) allowed us to study how the precatalyst structure influences the activation of the catalyst. We show that the activation time and mechanism depend on the tungsten loading: medium loading W/ZSM-5 catalysts (5 and 7 wt %) are easier to activate compared to low loading W/ZSM-5 catalysts (2 wt %), or high loading W/ZSM-5 catalysts (10 wt %). These insights could aid in developing suitable activation and regeneration protocols for W/ZSM-5 catalyst materials for the MDA reaction.

## Materials and Methods

### Catalyst Preparation

Zeolite H-ZSM-5 was obtained by calcining NH_4_-ZSM-5 (CBV 2314, Zeolyst) in static air at 550 °C for 7 h using a heating ramp of 2 °C/min. Before incipient wetness impregnation, H-ZSM-5 was dried for 4 h at 120 °C under vacuum in a round-bottom flask. A tungsten precursor stock solution was prepared by dissolving the appropriate amount of ammonium metatungstate hydrate (99.99%, Sigma-Aldrich) in ultrapure water in a volumetric flask. The stock solution was further diluted to obtain a solution with the desired concentration of tungsten for the various catalysts. Using a syringe, the amount of solution required to fill the pores of the zeolite (0.21 mL/g) was measured off and slowly dropped to the zeolite under vacuum. The obtained powder was dried overnight at 80 °C and calcined in static air at 550, 600, 650, or 700 °C for 7 h using a 2 °C/min heating ramp.

### Catalyst Characterization

Elemental analysis was carried out at Mikroanalytisches Laboratorium Kolbe, Germany, with an inductively coupled plasma (ICP) optical emission spectroscopy (OES) instrument (PerkinElmer, Waltham, MA, USA) after sample dissolution according to their standard in-house procedures.

Argon physisorption measurements were performed at −186 °C on a 3P 400 sync gas adsorption analyzer. Before the measurements, the samples were dried overnight at 400 °C under vacuum. The surface areas were calculated using the Brunauer–Emmett–Teller (BET) method. The total pore volume was determined at 0.99 *p*/*p*_0_.

Temperature-programmed desorption (TPD) with NH_3_ was performed using a Micrometrics Autochem 2910 apparatus. The amount of NH_3_ desorbed was determined using a thermal conductivity (TCD) detector. About 100 mg of material was loaded in a quartz reactor heated to 600 °C with a 10 °C/min ramp and held for 30 min under He flow to dry the sample. The sample was cooled to 100 °C and 20 pulses of NH_3_ (10 vol % in He) were applied to make sure all acid sites were saturated. Consequently, the sample was outgassed at 100 °C for 2 h and finally heated to 600 °C with a 5 °C/min ramp while the desorbed amount of NH_3_ was measured.

X-ray diffraction (XRD) patterns of the fresh samples were collected on a Bruker D8 in Bragg–Brentano mode using Cu K_α1,2_ radiation (λ = 0.15406 nm) and was operated at 40 kV and 40 mA. Data was collected while the sample was rotated at 15 rpm in the 2θ range from 5 to 55° with a 0.2° increment and scan speed of 0.5 s per step.

Raman spectra were recorded on a Renishaw InVia Raman microscope using 532 nm laser excitation through an ×50 objective and a 0.5 numerical aperture. Spectra were recorded between 100–1000 cm^–1^ with a 1200 l mm^–1^ grating, 10 s acquisition time, 50 accumulations, and a laser power output of 17.5 mW.

UV–vis Diffuse Reflectance Spectroscopy (DRS) was performed on a PerkinElmer Lambda 950 spectrophotometer equipped with an integrating sphere. The spectra were recorded from 800 to 200 nm with a 5 nm size step and an integration time of 0.4 s. A background was collected by placing a fresh ZSM-5 sample at the sample position.

Transmission Electron Microscopy (TEM) measurements were performed on a FEI Talos F200×. The microscope was operated at 200 kV and equipped with a high-brightness field emission gun (X-FEG) and a Super-X G2 energy dispersive X-ray (EDX) detector. The samples were deposited on a Formvar/Copper 300 mesh grid and were analyzed using scanning (S)TEM combined with high-angle annular dark-field (STEM-HAADF).

### Operando UV–Vis Diffuse Reflectance Spectroscopy

In a typical run, 300 mg of sample (212–425 μm) was loaded in a quartz fixed bed reactor with an internal diameter of 6.95 mm and was placed in a custom-built oven coupled to a thermocouple for temperature control. The sample was heated with a 5 °C/min ramp to 150 °C in the reaction mixture of 9 mL/min CH_4_ and 1 mL/min N_2_ (WHSV = 1.2 h^–1^, GHSV = 700 h^–1^) and held for 3 h as a reference point for the conversion calculations. After this, the reactor was heated to the reaction temperature with a 5 °C/min ramp under 10 mL/min N_2_; when this reaction temperature was achieved, the gases were switched to 9 mL/min CH_4_ and 1 mL/min N_2_ for 10 h. Some experiments involved a gaseous pretreatment prior to the MDA reaction. After reaching 700 °C, the sample was exposed to 10 mL/min N_2_ atmosphere for different times, or to 10 vol % H_2_ in N_2_ for 1 h.

Online activity and selectivity measurements were performed with an Interscience Compact gas chromatograph (GC) equipped with 3 flame ionization detector (FID) channels and 1 thermal conductivity detector (TCD) channel. Conversions were calculated according to
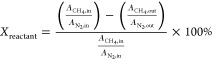
1where *A*_*i*,in_ (*i* = CH_4_ or N_2_) is the average integrated peak of three injections recorded at 150 °C before the reaction. Carbon yields were calculated according to
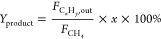
2where *F*_*i*_ denotes the molar flow of molecule *i* and *x* and *y* denote the number of carbon and hydrogen atoms in a product molecule.

UV–vis diffuse reflectance spectroscopy (DRS) was performed during the reaction using an AvaSpec2048L spectrometer equipped with a 100 μm slit connected to a high-temperature UV–vis optical fiber probe. The spectra were collected in reflection mode. A dark spectrum was recorded at room temperature and log(1/*R*) was calculated using
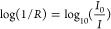
3where *I* is the intensity spectrum recorded during the reaction, and I0 is the first intensity spectrum recorded at reaction temperature.

### In Situ X-ray Diffraction

In situ X-ray diffraction (XRD) was performed with a Bruker Axs D8 Phaser Advanced instrument equipped with a Cu K_α1,2_ (λ = 1.54184 Å) source operating at 40 kV and 40 mA. An Anton Paar XRK900 Temperature Chamber was used to perform the in situ XRD experiments. For all experiments, the sample height was aligned so that the (270) reflection of ZSM-5 was detected at 32.4°. In a typical experiment, the sample was rotated and heated under a 100 mL/min N_2_ flow with a 5 °C/min ramp to 700 °C where the gas flow was changed to 10 mL/min H_2_ in 90 mL/min N_2_. At 700 °C, the sample was left for 5 h after which it was cooled down to room temperature in 100 mL/min N_2_. Diffractograms were recorded in the 2θ range of 32.4–42° with a 0.2° increment and a scan speed of 1.8 s per step.

## Results and Discussion

### Tungsten Loading Effect on Catalytic Performance

**Figure 1:**
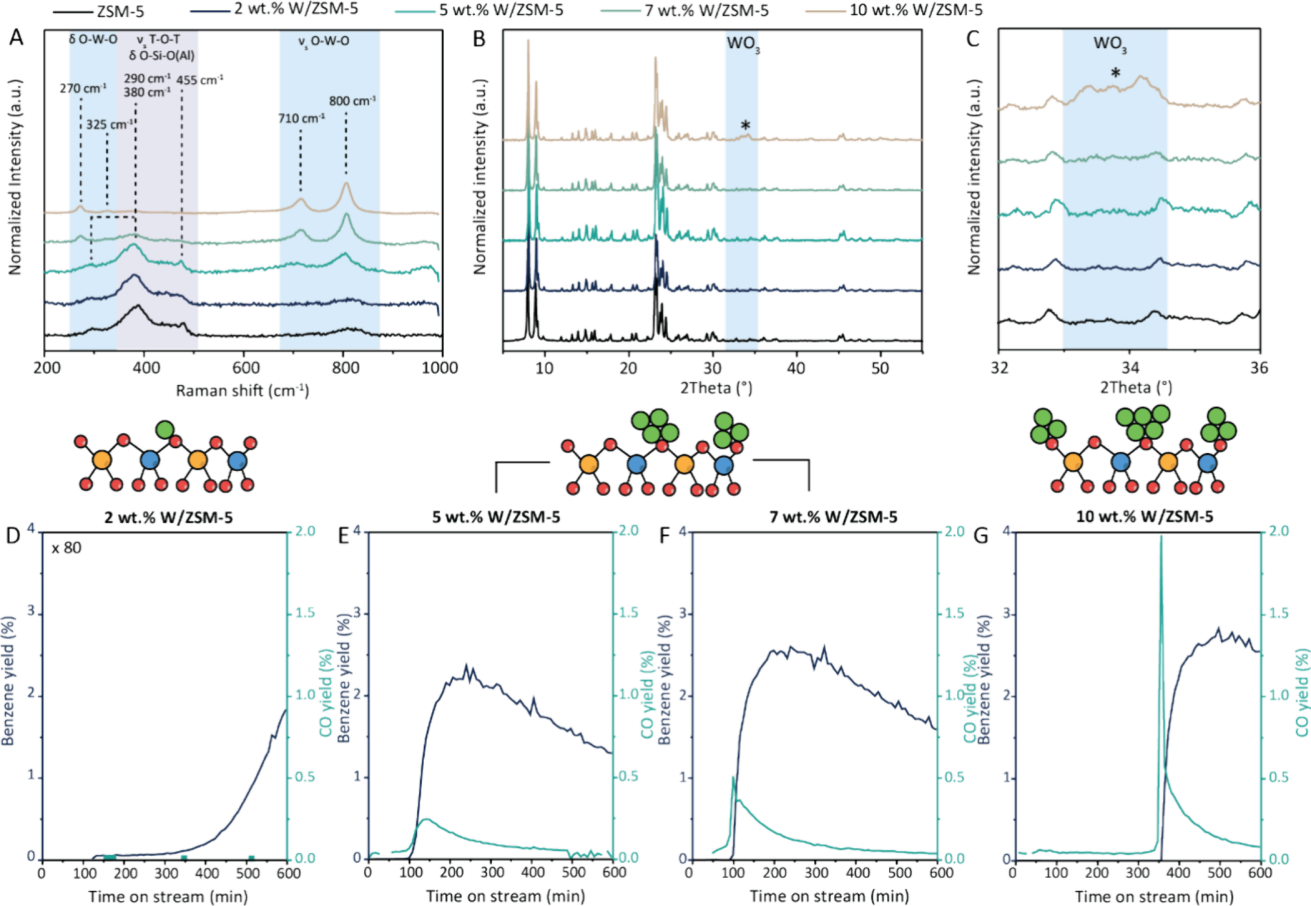
Characterization and activation of W/ZSM-5 precatalysts with different tungsten loading. (A–C) Ex situ characterization of the catalysts under study containing 2, 5, 7, and 10 wt % W/ZSM-5, after calcination at 550 °C: (A) Raman spectroscopy (B) X-ray diffraction (XRD) patterns, (C) zoom-in on the XRD pattern where WO_3_ reflection peaks appear. (D–G) Catalytic performance of the various W/ZSM-5 catalysts in the MDA reaction at 700 °C, in terms of benzene yield (blue) and CO yield (green) for (D) 2 wt % (×80), (E) 5 wt % (F) 7 wt %, and (G) 10 wt % W/ZSM-5. Conditions: 700 °C, 1 bar, 9 mL/min CH_4_, and 1 mL/min N_2_, 1.2 h^–1^ WHSV.

A series of W/ZSM-5 precatalysts was prepared with tungsten loadings of 2, 5, 7, and 10 wt % and calcined at 550 °C. The materials under study were characterized using Raman spectroscopy and X-ray diffraction (XRD), as summarized in Figure 1A–C. The Raman spectra of the 2 and 5 wt % W/ZSM-5 catalyst materials showed characteristic vibrations of the zeolite material: the peaks at ∼290, 380 cm^–1^ can be ascribed to the bending and the stretch mode of the tetrahedral-octahedral-tetrahedral (T-O-T) vibrations, respectively, whereas the peak at ∼455 cm^–1^ is assigned to the O–Si–O(Al) bending mode.^29^ For the 5 wt % W/ZSM-5 sample, two characteristic WO_3_ peaks were observed at ∼710 and ∼800 cm^–1^, which are assigned to stretching modes of the O–W–O vibration.^30,31^ For the 2 wt % W/ZSM-5 sample, the intensity of the O–W–O stretch vibration was very weak, indicating that for this sample, the tungsten was highly dispersed. For the 5, 7, and 10 wt % W/ZSM-5 materials, the intensity of the Raman bands for the O–W–O stretch vibrations was increasing. The presence of the O–W–O stretch vibration can be correlated to the size of the WO_*x*_ clusters; earlier work showed that this Raman peak is not present for particles smaller than 4 nm, whereas on a sample containing WO_*x*_ particles of 16 nm this Raman peak can be observed.^32^ On the 7 and 10 wt % W/ZSM-5 materials, additional Raman peaks were observed at ∼270 and ∼325 cm^–1^, which can be assigned to the O–W–O bending and deformation modes, respectively, and can be correlated to the presence of bigger WO_3_ particles.^32^ This shows that for the catalyst material with tungsten loadings of 7 and 10 wt %, larger tungsten oxide clusters are present compared to the samples containing 2 and 5 wt % tungsten, as expected. However, for the 7 and 10 wt % W/ZSM-5 samples, the presence monomeric species or smaller clusters cannot be excluded based on this data.

The effect of implementing higher W loadings was confirmed with X-ray diffraction (XRD), as shown in Figure 1B,C. For all materials, there was no change in the XRD reflection peaks that correspond to the zeolite material, showing that the crystallinity of the zeolite was not visibly impacted by W impregnation and subsequent calcination. For the 2, 5, and 7 wt % W/ZSM-5 samples, no additional reflection peaks in the XRD patterns could be observed, which indicates that the tungsten was highly dispersed or amorphous. For the 10 wt % W/ZSM-5 sample, however, an additional reflection peak was observed at ∼34°, which can be assigned to WO_3_ particles (Table S6). Using the DIFFRAC.EVA 5.1 software from Bruker, the particle size was estimated at ∼10 nm by applying the Scherrer equation on the peak between 33 and 34.8°.^33^ Based on the knowledge gained from the Raman and XRD measurements we can get an indication of the dispersity of the tungsten on the precatalysts. For the high loading case, we can observe the bulk-like WO_3_ band (∼10 nm), but this observation does exclude the presence of monomeric species and/or clusters. The same holds for the 5 and 7 wt % precatalysts; here, we can detect the smaller WO_3_ clusters, but the presence of monomeric species cannot be excluded. For the 2 wt % sample, we did not observe signals from tungsten species with Raman or XRD, suggesting that the 2 wt % W/ZSM-5 catalyst is highly dispersed. To get a further indication of dispersity, we calculated the W/Al ratio based on ICP-OES measurements (Table S2). These show that in the 2 wt % sample, the W/Al atomic ratio was 1:2. However, in the case of 5 and 7 wt % W precatalysts, respectively 1.5 and 2 tungsten atoms are present per Al site, and in the case of the 10 wt % sample, 3.5 tungsten atoms are present per Al sites. These findings suggest that there is a correlation between tungsten loading and dipersity, but the presence of some highly dispersed species in mid and high loading samples cannot be excluded based on this data. We performed additional ex situ UV–vis Diffuse Reflectance Spectroscopy (DRS) measurements on the calcined catalyst materials to confirm this trend. In Figure S1, the spectra as well as the Tauc plots are shown, and in Figure S2 the band gap values (*E*_g_) obtained from the Tauc plots of the materials under study are shown. We observe decreasing band gap values with increasing tungsten loading. Watanabe et al. showed the relation between the band gap value and the particle size; bulk WO_3_ particles have smaller *E*_g_ values (∼2.6 eV) than smaller particles (∼3.4 eV).^34^ This trend is in line with our measurements, indicating the presence of large particles in the samples with higher tungsten loadings and smaller particles in the low loading samples. These insights show there is a correlation between tungsten loading and dispersity, but we cannot exclude the presence of certain species based on this data.

The catalytic performance in the MDA reaction of the 550 °C-calcined W/ZSM-5 materials was studied at 700 °C, atmospheric pressure, and 1.2 h^–1^ WHSV (Figure 1D–G). In the Supporting Information, the methane conversions for all tested materials as well as other product yields are shown (Figures S14–S34). While testing catalysts at temperatures higher than the calcination temperature is unusual, we have purposely chosen this method to follow the activation of the precatalyst under reaction conditions. Higher calcination temperatures are discussed in the next section. Figure 1D–G show the benzene yields and the CO yields for the W/ZSM-5 catalysts with 2, 5, 7, and 10 wt % tungsten loading. The materials showed different catalytic behavior: the 2 wt % W/ZSM-5 catalyst, of which the catalytic results 80× magnified for comparison, produced negligible amounts of benzene and was practically inactive for the MDA reaction (Figure 1D). The inactivity of the low W loading catalyst seems to be related to the dispersion of the tungsten over the zeolite ZSM-5 material, as shown by Raman and XRD results. This observation is supported by literature. More specifically, Çağlayan et al. showed in a density functional theory study that the reduction of monomeric tungsten species, believed to be involved in catalyst activation, is less spontaneous compared to the reduction of dimeric species,^16^ which would explain the inactivity of the 2 wt % W/ZSM-5 sample.

On the other hand, the 5, 7, and 10 wt % W/ZSM-5 materials showed considerable benzene yields. However, these materials required a long activation time. Benzene production started 100 min after CH_4_ exposure in the case of the 5 and 7 wt % W/ZSM-5 catalyst materials, while for the 10 wt % W/ZSM-5 catalyst material, it took 350 min before benzene was observed. In the MDA process, catalyst activation is commonly believed to be linked to the formation of (oxy)carbide metal species, which results in the production of CO during the activation period.^35,36^ However, CO was only formed after the first 100 min (for the 5 and 7 wt % W/ZSM-5 catalysts) or 350 min (for the 10 wt % W/ZSM-5 catalyst), almost simultaneously with benzene formation. This indicates that the carburization process did not (sufficiently) occur during the activation time, and suggests that another phenomenon is possibly involved in the formation of the active site for methane dehydroaromatization.

Based on the observations from the catalyst characterization and the catalytic performance tests, we divided the studied catalyst materials into three showcases: the low weight loading case (i.e., 2 wt % W/ZSM-5); the medium weight loading regime (i.e., 5 and 7 wt % W/ZSM-5); and the high weight loading case (i.e., 10 wt % W/ZSM-5). Since no activity was observed in the low weight loading case, the remainder of this paper will focus on the activation mechanism of the W/ZSM-5 catalysts in the medium and high weight loading regimes.

### Catalyst Activation by Tungsten Redispersion at High Temperature

**Figure 2:**
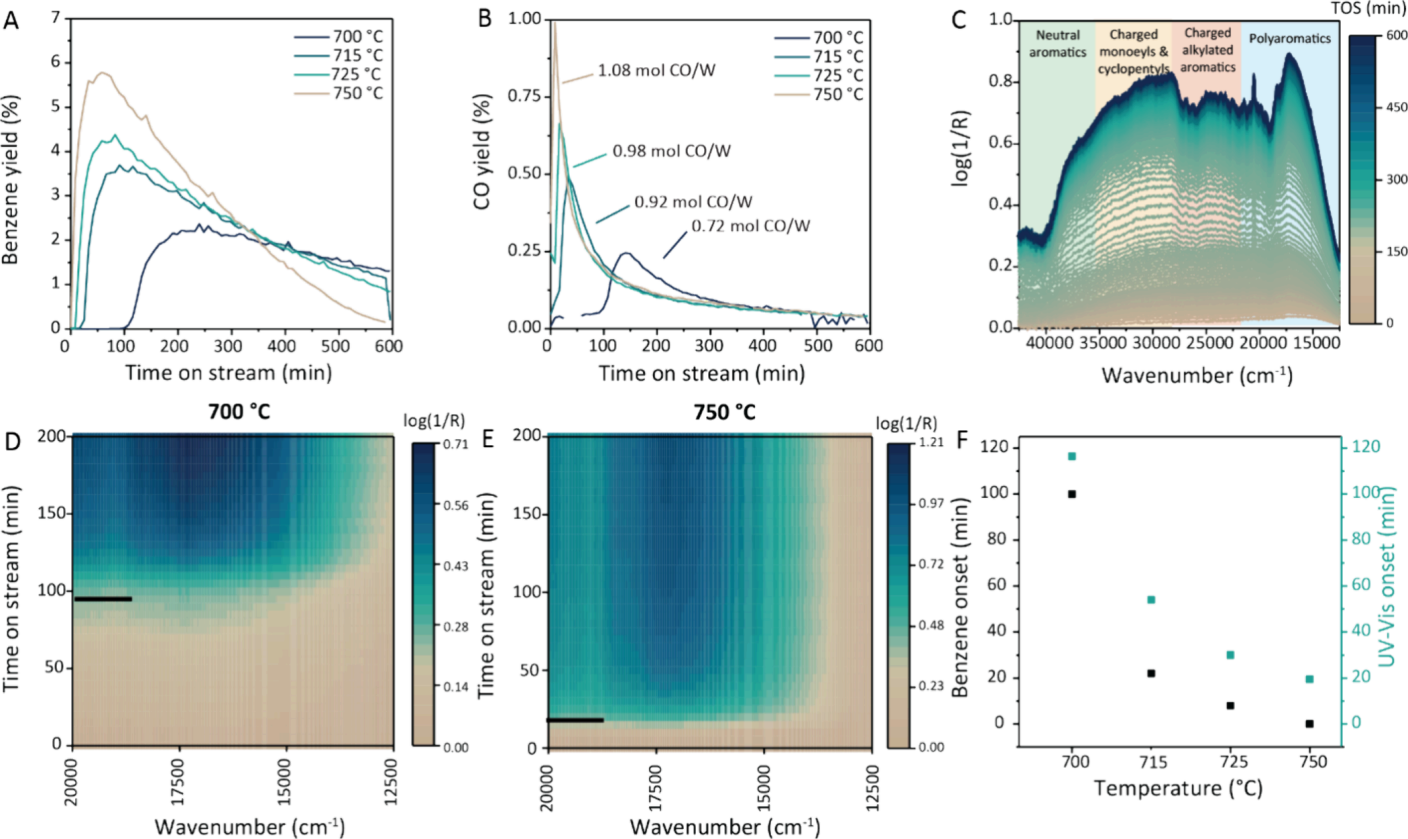
Effect of reaction temperature on the activation period of 5 wt % W/ZSM-5 studied by operando UV–vis spectroscopy. (A, B) Catalytic performance of 5 wt % W/ZSM-5, calcined at 550 °C, at different reaction temperatures in the 700–750 °C range, expressed as (A) benzene yield and (B) CO yield, including the amount of CO released per W. The stoichiometric amount of WC formation would be 3 CO/W. (C) Operando UV–vis spectra recorded during a catalytic experiment at 700 °C. (D, E) Contour plots of the operando UV–vis spectra at 700 and 750 °C respectively, showing the earlier onset of carbon deposition (indicated with the black line) with higher reaction temperature. (F) Correlation between the onset of benzene formation and onset of the 17260 cm^–1^ peak in the UV–vis spectra as a function of reaction temperature. Conditions: 1 bar, 9 mL/min CH_4_ and 1 mL/min N_2_, 1.2 h^–1^ WHSV.

To gain a better understanding of the activation behavior in the medium W loading regime, the performance of the 5 wt % W/ZSM-5 catalyst was tested as a function of reaction temperatures, at 700, 715, 725, and 750 °C, in an operando UV–vis spectroscopy setup. In Figure 2A, the benzene yields during the MDA reaction at different reaction temperatures are shown. By increasing the reaction temperature to 715, 725, and 750 °C, the time before the onset of benzene production was shortened from 100 min to 22, 8, and 0 min, respectively. The onset of CO production, shown in Figure 2B, coincided with the start of benzene formation, indicating that during the observed activation period, WO_*x*_ carburization did not take place, or that it only happened to a very limited extent, producing CO below the detection limit of our equipment. The molar ratio of CO/W was calculated through integration of CO production over time, and based on the W weight loading and catalyst weight. The values of CO/W molar ratio varied between 0.7 and 1.1 for the different catalytic tests. Assuming WO_*x*_ carburization according to
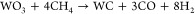
4

the CO/W molar ratio for complete W carburization would be 3. The results thus indicate that the MDA active site under the investigated conditions was not a fully carburized tungsten carbide, but more likely a tungsten (oxy)carbide.

Since carbon deposition was also proposed as a way for catalyst activation in the MDA reaction in a catalytic carbon-pool mechanism, we investigated the carbon build-up on the catalyst surface during the activation and reaction periods using operando UV–vis spectroscopy. In Figure 2C, the operando UV–vis spectra over time during the MDA reaction at 700 °C are shown. A growth in absorbance over time in the regions corresponding to neutral aromatics (i.e., 38,000–35,000 cm^–1^), hydrocarbon pool intermediates (i.e., 35,000–30,000 cm^–1^), and polyaromatic hydrocarbons (i.e., below 25,000 cm^–1^) can be observed (see Table S4 for a complete overview of the band assignments of hydrocarbon species).^37^ Interestingly, in the first 100 min of the MDA reaction at 700 °C there was only a very minimal and slow increase in UV–vis absorbance, as shown by the contour plot of the spectra recorded for the reaction and the spectra recorded during the activation period (Figures 2D and S4). Only once the catalyst started producing benzene after 100 min, the UV–vis absorbance increased at a rate as would be expected during the MDA process.^37^ When the reaction is carried out at 750 °C, the benzene production starts immediately, and also the UV–vis absorbance (Figure 2E) increases at an earlier time on stream. This trend was observed across the various reaction temperatures measured, which is summarized in Figure 2F. In all cases, only a minimal increase in absorbance is observed during the activation period (Figure S4) and the rapid increase in absorbance coincides with the production of aromatics.

The apparent activation energy (*E*_a_) for the activation of the 5 wt % W/ZSM-5 catalyst could be roughly estimated using the Arrhenius equation, considering that the ratio between two activation times (*t*_1_/*t*_2_) at two temperatures *T*_1_ and *T*_2_ is inversely proportional to the rate constant of the activation reaction (*k*_2_/*k*_1_):
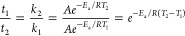
5

with *R* being the gas constant, and *A* the pre-exponential factor. In eq 5 we assumed the *A* and *E*_a_ to be constant at the different temperatures considered. Knowing the reaction temperatures (i.e., 700, 715, and 725 °C) and the time at which benzene evolution was first observed (i.e., 100, 22, and 8 min), we can derive the apparent *E*_a_ for each combination of two temperatures (see Figure S12 and Table S7 for details). The resulting apparent *E*_a_ had a very high value of >700 kJ/mol. Such high activation energies have been reported for processes, such as sintering and phase transformations.^38,39^ This observation, in combination with the observation that the activation is not accompanied by the formation of CO, indicates that the activation of the W/ZSM-5 catalyst is due to a thermally activated structural change of the catalyst, and not chemically induced by reaction with CH_4_.

**Figure 3:**
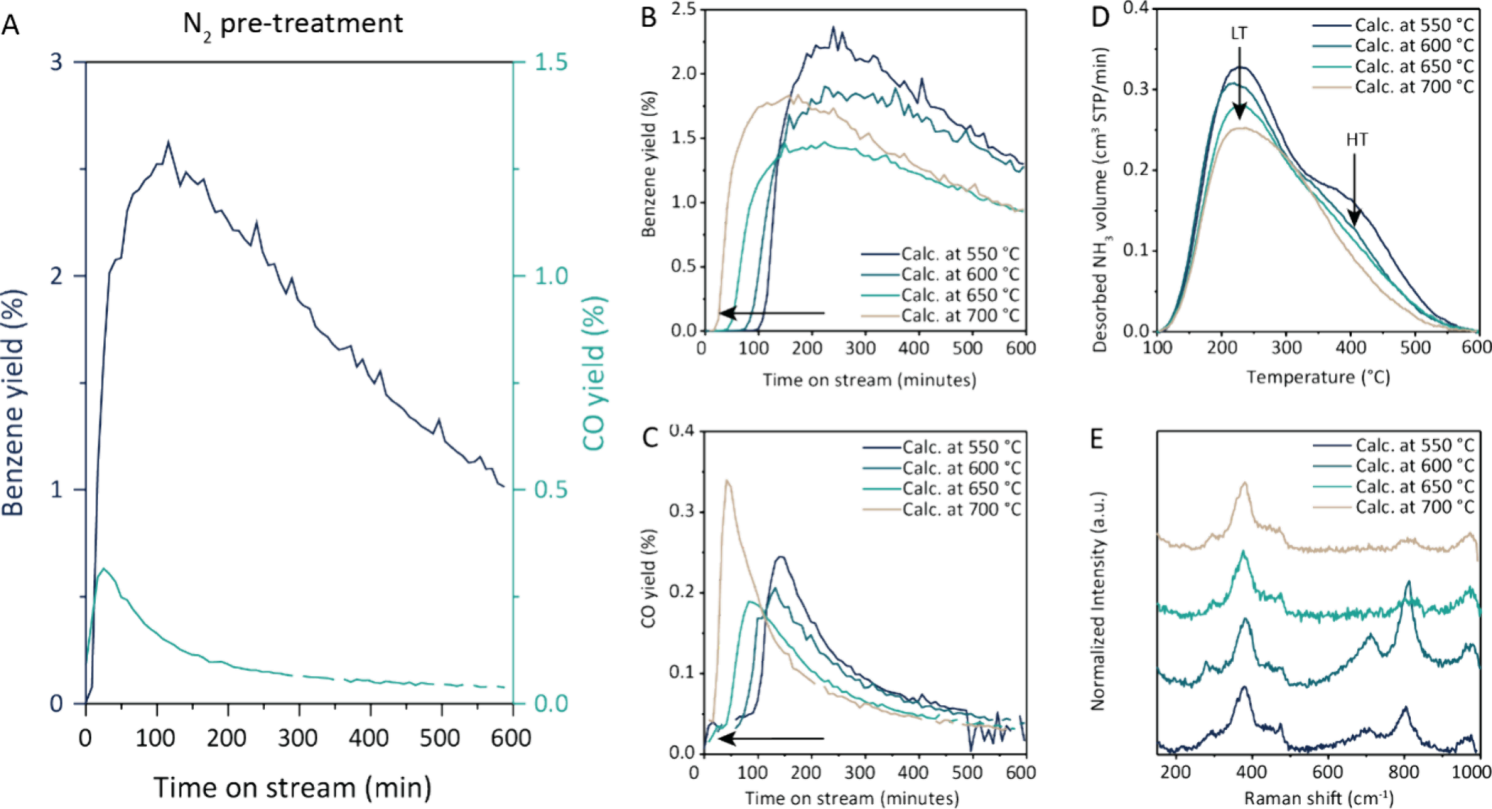
Evidence for thermally activated tungsten redispersion during catalyst activation. (A) Benzene and CO yield during the methane dehydroaromatization (MDA) reaction at 700 °C, for a 5 wt % W/ZSM-5 catalyst calcined at 550 °C and pretreated in N_2_ at 700 °C for 100 min, (B, C) benzene and CO yield for the 5 wt % W/ZSM-5 catalysts calcined at 550, 600, 650, and 700 °C. Conditions: 700 °C, 1 bar, 9 mL/min CH_4_ and 1 mL/min N_2_, 1.2 h^–1^ WHSV. (D, E) Characterization of the 5 wt % W/ZSM-5 precatalysts calcined at 550, 600, 650, and 700 °C, where (D) the NH_3_-temperature-programmed desorption (TPD) curves show two peaks at low temperature (LT) and high temperature (HT), and (E) ex situ Raman spectroscopy shows the disappearance of O–W–O peaks after calcination at high temperatures.

To test the hypothesis that catalyst restructuring was involved in the activation of the 5 and 7 wt % W/ZSM-5 catalyst materials, we performed a N_2_ pretreatment for 100 min at 700 °C before switching to CH_4_ (Figures 3A and S20). For the 5 wt % W/ZSM-5 catalyst, production of benzene and CO was observed as soon as CH_4_ was introduced to the reactor after the N_2_ pretreatment. The N_2_ treatment also helped to reduce the length of the activation period for the 7 wt % catalyst by 20 min (Figure S26). This excludes the role of CH_4_ in activating the catalyst during the first 100 min, and shows that before the formation of the active phase, a thermally induced structural change of the catalyst is taking place.

To further investigate the thermal effects on the W active phase, three other 5 wt % W/ZSM-5 catalysts were prepared at calcination temperatures of 600, 650, and 700 °C, and tested in the MDA reaction at 700 °C. The benzene and CO yields over time for such catalyst materials are shown in Figure 3B,C. With increasing calcination temperatures, the observed activation period became increasingly shorter, showing that with an adjustment in the preparation method, we can achieve the same effect as with an N_2_ pretreatment. This indicates that a thermally induced restructuring of the catalyst is taking place during activation of the 5 and 7 wt % W/ZSM-5 catalysts. Furthermore, there is a striking difference when comparing the operando UV–vis spectra recorded during the N_2_ pretreatment to the spectra recorded during the activation period where the catalysts calcined at various temperatures are exposed to CH_4_ (Figure S6). When the catalysts are exposed to CH_4_, a UV–vis band between 35,000 and 30,000 cm^–1^ appears during the activation period, indicating hydrocarbon pool-like species are deposited on the surface during activation. However, the activity is still zero, indicating the formation of these species does not play a role in the activation process and another, slower activation process is taking place. The band corresponding to hydrocarbon pool species does not appear during the activation in N_2_ due to the absence of carbon in the feed, but after this pretreatment, the catalyst shows instant activity. This observation confirms that the formation of hydrocarbon pool species is not the rate-limiting step in the activation of W/ZSM-5 catalysts, and another, slower process is happening.

To understand how the calcination temperature influences the precatalyst structure, and how this results in the shortened activation time, the 5 wt % W/ZSM-5 precatalysts prepared with various calcination temperatures were characterized using NH_3_-TPD and Raman spectroscopy, shown in 3D,E. The NH_3_-TPD data showed a decrease in the low-temperature (LT) and high-temperature (HT) NH_3_ peaks with increasing catalyst calcination temperature (Figure 3D). The observed lower Bro̷nsted acidity after higher calcination temperature can be explained by at least two hypotheses, which do not exclude one another: (i) a higher fraction of Bro̷nsted acid sites were occupied by tungsten, consistent with a thermally induced WO_*x*_ redispersion on the ZSM-5; or (ii) framework aluminum was partially removed from the zeolite framework at higher calcination temperatures.^40^ To determine which effect is dominant, a ZSM-5 sample was calcined at 700 °C and analyzed with NH_3_-TPD. For all samples prepared with different loadings and at varying calcination temperatures, we plotted the total amount of NH_3_ adsorbed per gram sample as a function of tungsten loading (Table S3, Figure S3). Both higher W loadings as well as higher calcination temperatures resulted in a reduction in the number of Bro̷nsted acid sites, which could be reducing the performance as the BAS are hypothesized to be active sites.^41^ However, higher calcination temperatures resulted in a bigger loss of acid sites than implementing higher tungsten loadings, since significantly lower benzene yields were observed when implementing higher calcination temperatures.

This was observed in the performance as well as higher calcination temperatures lead to Additionally, we have performed ex situ Raman spectroscopy on the 5 wt % W/ZSM-5 precatalysts calcined at different temperatures (Figure 3E). For the catalysts calcined at 550 and 600 °C, sharp Raman peaks at ∼710 and ∼ 800 cm^–1^ were observed, which are attributed to the O–W–O stretch vibration.^30,31^ These peaks were reduced in intensity for the catalysts calcined at 650 and 700 °C, which could indicate a higher dispersion of tungsten dispersion over the catalyst support when calcined at higher temperatures. Similar results were obtained for the 7 wt % W/ZSM-5 as a function of calcination temperature (Figures S9 and S25). Our ex situ UV–vis DRS measurements also point in the direction of redispersion as calcination at 700 °C instead of 550 °C for the 5 and 7 wt % W/ZSM-5 samples results in higher band gap values, indicating the presence of relatively smaller WO_3_ particles with increasing calcination temperature (Figures S1 and S2).^34^ This is in line with a previous study where a higher dispersion of tungsten over the support was observed after calcination at higher temperatures.^42^ However, we cannot confirm the presence of smaller clusters with these techniques since it is below the detection limit of our equipment. Nonetheless, we can conclude that the calcination temperature influences the tungsten structure.

The combined insights from the various experiments carried out with the 5 and 7 wt % W/ZSM-5 catalysts indicate redispersion of tungsten over the zeolite during the activation period for catalysts in the medium loading regime. The redispersion is thermally induced and facilitates the formation of the active site for the MDA reaction. Nonetheless, based on these results we cannot guarantee that the catalysts pretreated in N_2_, or prepared at higher calcination temperatures, or activated in CH_4_ undergo a similar structural evolution during the reaction. Therefore, the 5 wt % W/ZSM-5 catalyst material calcined at 550 and 700 °C were studied with STEM-HAADF. In Figure S11, the images of the fresh materials are compared to the materials that have been (1) exposed to CH_4_ for 45 min at 700 °C, and (2) that are spent, after 10 h of reaction. In the case of the fresh materials, white structures along the edges of the ZSM-5 crystal can be observed which are the tungsten species. After having been exposed to CH_4_ for 45 min at 700 °C, these white structures cannot be observed anymore. This shows that during the activation period the tungsten is redispersing. After 10 h of reaction these structures are also not present. The similarities indicate that the species undergo a similar structural evolution, but to confirm this, operando X-ray studies at synchrotron radiation facilities are required, which is beyond the scope of this work.

Interestingly, in the case of the 2 wt % W/ZSM-5 catalyst, where the W dispersion was expected to be the highest, as was shown with both Raman and UV–vis DRS measurements (Figures S1 and S9) a higher calcination temperature did not result in catalyst activation (Figure S15). This suggests one W atom needs to be near other W atoms to be active in the MDA reaction. As shown with ICP-OES (Table S2) in the medium loading case, theoretically more than one tungsten atom is available per Al site, making it more likely that two W atoms are close together.

### Tungsten Redispersion and Activation by Reduction at High Tungsten Weight Loading

As shown in the case of 5 and 7 wt % W/ZSM-5 catalyst materials, W redispersion is correlated with catalyst activation during the MDA reaction. Accordingly, it can be expected that at higher W loadings, where W redispersion may be hindered by surface coverage effects, the activation would take longer. To study this, two 10 wt % W/ZSM-5 catalysts were prepared and calcined at 550 and 700 °C. The presence of WO_*x*_ particles was confirmed with both Raman spectroscopy and XRD (Figures S9D and S10). After calcination at 700 °C, the characteristic O–W–O vibrational modes (i.e., ∼270, 325, 710, and 800 cm^–1^) were still observed. Moreover, in the XRD pattern of the 10 wt % W/ZSM-5 sample calcined at 700 °C, the reflection peak at ∼34° was also still present, indicating the presence of WO_3_ particles of about 10 nm. Also, the *E*_g_ values obtained from the UV–vis DRS measurements barely changed for the two calcination temperatures, indicating that the large WO_3_ particles did not redisperse after calcination at 700 °C. However, based on these results, it cannot be excluded that small clusters and/or monomeric W species were also formed and are present in the sample together with the WO_3_ particles.

**Figure 4:**
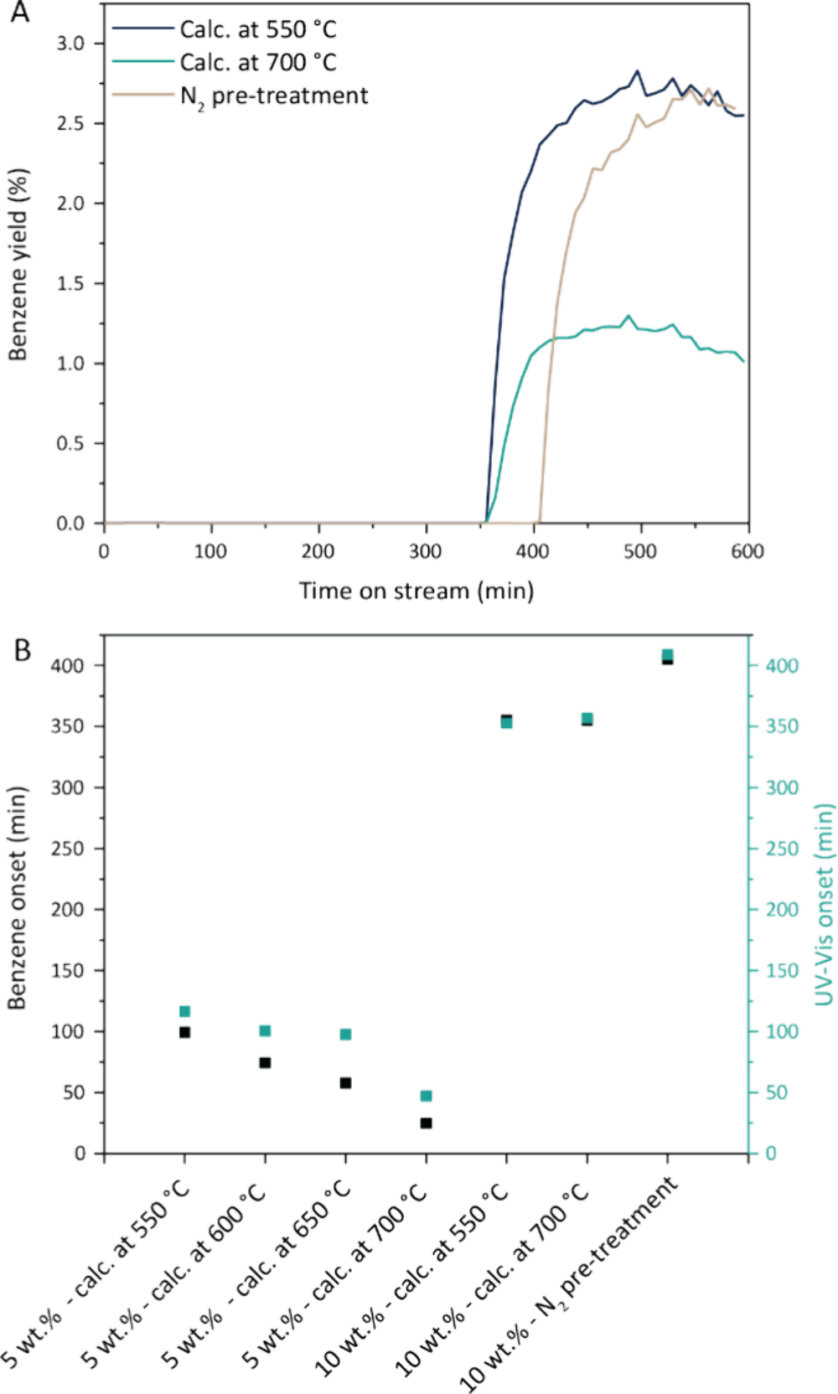
Hindered thermal activation of 10 wt % W/ZSM-5 methane dehydroaromatization (MDA) catalysts. (A) MDA catalytic performance of 10 wt % W/ZSM-5 as a function of calcination temperature and a N_2_ pretreatment at 700 °C for 350 min (MDA conditions: 700 °C, 1 bar, 9 mL/min CH_4_ and 1 mL/min N_2_, 1.2 h^–1^ WHSV). (B) Comparison of the effect of calcination temperature on the product onset for 5 wt % W/ZSM-5 compared to the 10 wt % W/ZSM-5.

In line with these findings, the activation of 10 wt % W/ZSM-5 calcined at 550 °C required exposure to CH_4_ for even longer times compared to the 5 and 7 wt % W/ZSM-5 counterparts (350 vs 100 min), indicating that either redispersion of tungsten takes longer for higher weight loadings, or that other activation mechanisms are at play (Figure 4A). This was confirmed when the 10 wt % W/ZSM-5 catalyst calcined at 700 °C was tested in the MDA reaction. An increase in calcination temperature did not result in a shorter activation time (Figure 4A), which would be expected from the Raman spectroscopy and XRD results as we did not observe the disappearance of the WO_3_ peaks. The activation time remained 350 min, and the benzene yield was halved, due to the loss of Bro̷nsted acid sites of the zeolite material (Table S3, Figure S3).

Additionally, we performed an N_2_ pretreatment for 350 min at 700 °C on the 10 wt % W/ZSM-5 sample and consequently tested it in the MDA reaction (Figure 4A). Notably, the N_2_ pretreatment did not result in a shorter activation period as observed for the 5 and 7 wt % W/ZSM-5 samples. In Figure 4B the effect of calcination temperature on the product onset for the 5 and 10 wt % W/ZSM-5 catalysts are summarized. Whereas for the 5 wt % W/ZSM-5 catalyst series a clear effect of calcination temperature is observed, for the 10 wt % W/ZSM-5 catalysts, no change is observed. The fact that the calcination temperature and the N_2_ pretreatment did not result in shorter activation times shows that the activation mechanism for the 10 wt % W/ZSM-5 catalyst likely does not proceed by redispersion of tungsten over the zeolite. This was also confirmed with ex situ Raman on the 10 wt % W/ZSM-5 calcined at 700 °C. Additionally, no change in band gap energy was observed with ex situ UV DRS measurements (Figure S2), showing that the tungsten particles are not redispering. This could possibly be explained because there are not enough anchoring points for W redispersion, as confirmed with ICP-OES, which shows that ∼3.5 W atoms are present per Al site (Table S2), or because migrating W species are adsorbed by W nanoparticles in an Ostwald ripening process.

**Figure 5:**
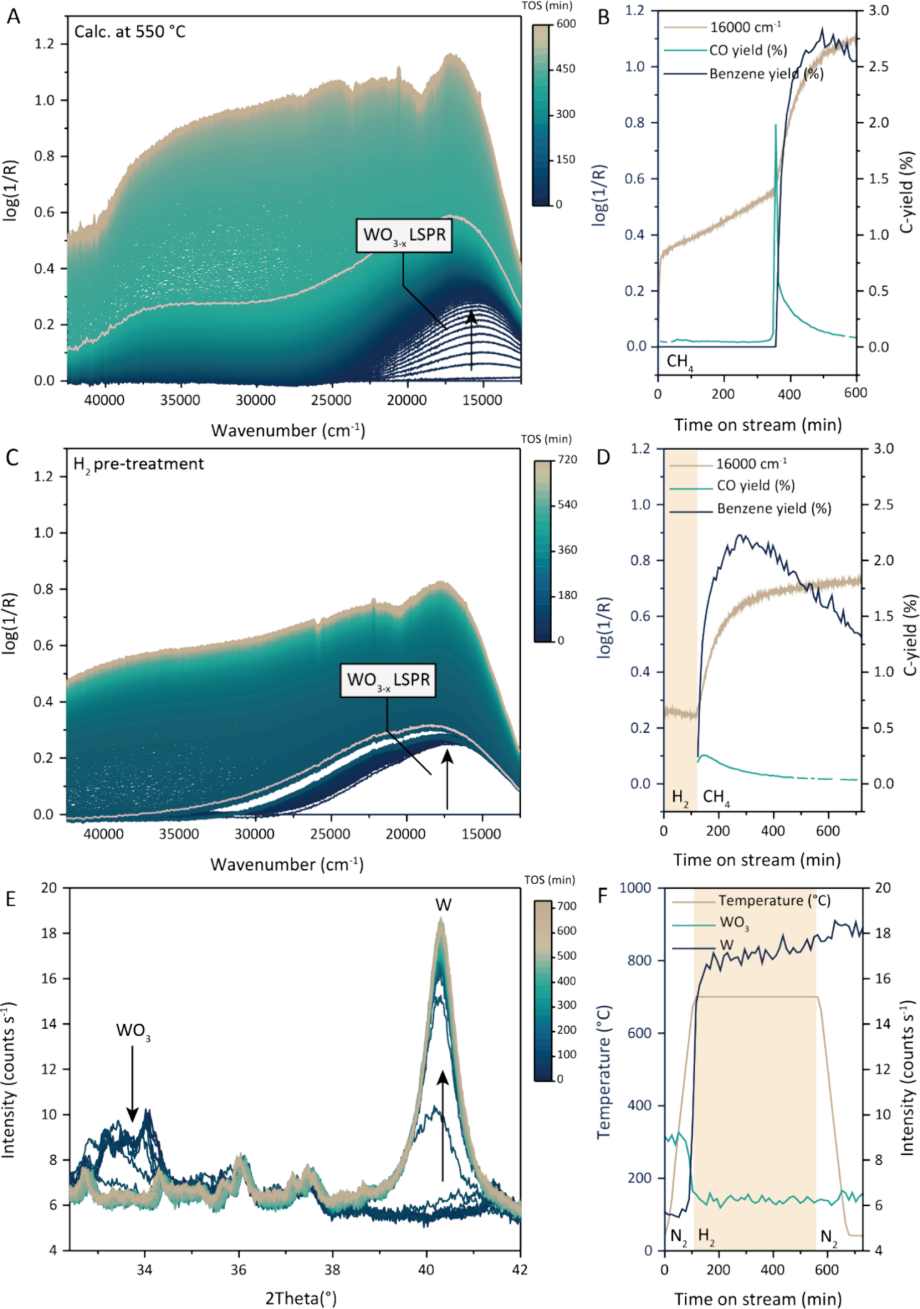
Activation of high loading tungsten catalyst via WOx reduction. (A) Operando UV–vis spectra recorded at 700 °C under methane dehydroaromatization (MDA) conditions for the 10 wt % W/ZSM-5 catalyst calcined at 550 °C with the pink spectrum indicating the time at which benzene production starts, and (B) the respective benzene and CO yield, with the evolution of the UV–vis band intensity at ∼16,000 cm^–1^ over time. (C) Operando UV–vis spectra of the 10 wt % W/ZSM-5 catalyst recorded at 700 °C during a 100 min H_2_ pretreatment and under MDA conditions with the pink spectrum indicating the time at which we switch to CH_4_ and the start of the benzene production. (D) Respective time evolution of the intensity of the UV–vis band at ∼16,000 cm^–1^, together with benzene and CO yield in the MDA reaction. (E) In situ X-ray diffraction (XRD) patterns of 10 wt % W/ZSM-5 (calcined at 550 °C) during H_2_ reduction at 700 °C, and (F) the respective time evolution of the reflection peaks at ∼34°, belonging to WO_3_, and at ∼40° belonging to metallic W as well as the temperature profile during the in situ experiment.

To understand how the 10 wt % W/ZSM-5 activates under MDA conditions, we studied the system using operando UV–vis spectroscopy. In the UV–vis spectroscopy time series recorded during the reaction (Figure 5A), an absorption band at ∼16,000 cm^–1^ appears which shifts to slightly higher wavenumbers over time. This band was previously attributed to the formation of polyaromatic species on the catalyst surface. However, when we plot the intensity of this absorption band over time, together with the benzene yield and CO yield (Figure 5B), this absorption band grows already before product formation. In literature, this absorption band was assigned to the localized surface plasmon resonance (LSPR) of partially reduced WO_3_ nanoparticles (Table S4).^43−46^ This suggests that at higher tungsten loadings, reduction, and not redispersion, was the rate-limiting step in the formation of the active site for the MDA reaction.

To confirm this hypothesis, we performed a H_2_ pretreatment at 700 °C prior to switching to MDA conditions. During the activation in H_2_, the absorption band at ∼16,000 cm^–1^ appeared, and indeed, after switching to CH_4_, the catalyst was immediately active, showing reduction is the activation mechanism for the high loading case (Figure 5C,D). Similar to the medium loading case, also here the formation of a hydrocarbon pool is not the rate-limiting step. When comparing the UV–vis spectra recorded during the activation in CH_4_ to the spectra recorded during the H_2_ pretreatment (Figure S7) the band corresponding to hydrocarbon pool species appears between 35,000 and 30,000 cm^–1^ for the samples activated in CH_4_, which is absent during the H_2_ activation. Since the H_2_-activated sample shows immediate activity, this confirms that the formation of the hydrocarbon pool species is not the limiting factor in the activation process.

However, it is intriguing that in the case of the 10 wt % W/ZSM-5 samples activated in CH_4_ the formation of CO, indicating the transformation of the tungsten oxide to the tungsten (oxy)carbide and thus the reduction of the precatalyst, only starts after the activation period where we assume the tungsten is reduced. One possible explanation for this is that CO is released in small amounts below the detection limit of our equipment.

Another explanation is that the reduction of WO_3_ to form WO_3-x_ is a thermal process. When inspecting the operando UV–vis spectra recorded during the activation period of the 10 wt % W/ZSM-5 catalysts (Figure S7), a band at ∼16,000 cm^–1^ appears in the cases where we activate in CH_4_, or perform a pretreatment in N_2_ or H_2_. It is intriguing that the formation of this band also happens during the pretreatment in N_2_, as the pretreatment does not result in immediate activity. Upon closer inspection, however, the spectra recorded during the activation in CH_4_ or H_2_ show a blue shift, while the peak does not shift during the N_2_ pretreatment. The blue shift indicates an increase in oxygen vacancies, and thus a higher reduction degree of WO_3._^43^ This would also mean that during the N_2_ pretreatment, some oxygen vacancies are generated in a thermal reduction process, but not enough to form the active phase.

We could confirm the reduction of WO_3_ to metallic W in H_2_ atmosphere with in situ XRD. In Figure 5E,F, a 2D plot of the XRD patterns and a plot showing the temperature behavior of the WO_3_ and W peak during the reduction of 10 wt % W/ZSM-5 calcined at 550 °C can be found, respectively. Before the reduction, the WO_3_ reflections are visible at ∼34°. At 700 °C, the atmosphere was switched to 10 vol % H_2,_ and the reflection peak at ∼34° disappeared almost immediately and was accompanied by the fast growth of a reflection peak at ∼40°, which corresponds to metallic tungsten, as shown in Figure 5F.^47^ After 5 h of reduction, the sample was cooled down in N_2_ and the reflection peak at ∼40° remained stable. From this, we can conclude that WO_3_ particles were reduced to W in an H_2_ atmosphere. We suggest that the active phase for the MDA reaction is more easily formed starting from partially reduced WO_*x*_ or metallic W, resulting in immediate MDA activity after switching from H_2_ to CH_4_.

To further substantiate the distinct activation mechanism for the high W loading precatalyst, we performed additional catalytic performance tests for a 10 wt % W/ZSM-5 catalyst in the temperature range between 700 and 750 °C (Figure S13) to calculate the apparent activation energy using the same method as was done for the 5 wt % W/ZSM-5 catalyst. A new batch of 10 wt % W/ZSM-5 catalyst was prepared using the incipient wetness impregnation method, which resulted in an induction time of 200 min when MDA was performed at 700 °C, compared to 350 min for the first catalyst batch. The average apparent activation energy that was calculated was ∼260 kJ/mol (Table S8), which is in line with earlier reported activation energies for the reduction of tungsten with carbon, as opposed to the much higher activation energy for tungsten redispersion.^48^ This further confirms the different activation mechanism for 10 wt % W/ZSM-5 catalysts, where thermal activation is not sufficient to yield an active catalyst.

**Scheme 1:**
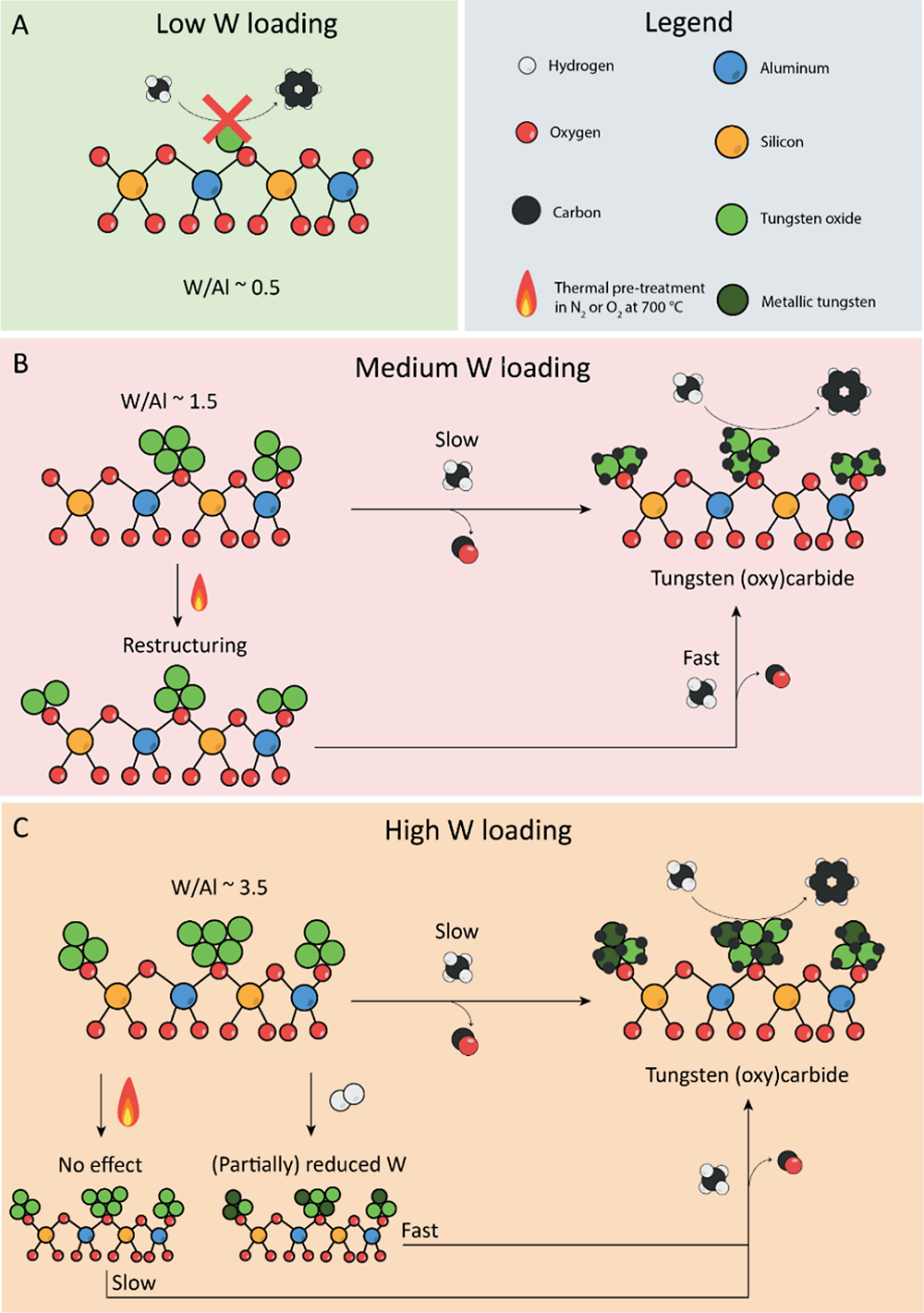
Understanding Loading Effects on the Activation Mechanism of W/ZSM-5 Catalysts for the Methane Dehydroaromatization (MDA) Reaction Model for the activation mechanism of W/ZSM-5 for different tungsten weight loadings, based on the observed catalytic, X-ray diffraction (XRD), Raman and UV-vis spectroscopic results. (A) In the low (i.e., 2 wt.%) W weight loading case, the tungsten oxide cannot be activated in CH_4_ at 700 °C, resulting in an inactive MDA catalyst. (B) For the medium (5–7 wt.%) W weight loading regime, the activation of the tungsten oxide species under MDA conditions is slow, and involves the thermally-activated redispersion of tungsten oxides over the zeolite surface. (**C**) In the high (i.e., 10 wt.%) W weight loading case, the tungsten oxide present on the precatalyst material, require several hours under MDA conditions to become active. In this case, thermal pretreatments in N_2_ or O_2_ do not lead to a faster activation. Instead, the activation proceeds by (partial) reduction of CH_4_. Accordingly, an H_2_ pretreatment at 700 °C resulted in immediate MDA activity when switching to CH_4_.

The findings of this study are summarized in Scheme 1. We showed that the dispersion of tungsten over zeolite ZSM-5 plays an important role in the activation of the corresponding MDA catalyst: in the low weight loading case (i.e., 2 wt % W/ZSM-5), the tungsten was likely present as monomers that cannot be activated, resulting in inactivity in the MDA reaction. In the medium weight loading regime (i.e., 5 and 7 wt % W/ZSM-5), considerable benzene yields are observed, but it took ∼100 min before MDA activity was observed. During the activation period, the tungsten was redispersed. This process was thermally induced since shorter activation times were observed after a N_2_ pretreatment, or when the catalyst was prepared at higher calcination temperatures. Finally, at high weight loadings (i.e., 10 wt % W/ZSM-5), it took ∼350 min before MDA activity was observed. The activation time could not be shortened through a thermal treatment in either air or an inert atmosphere. This shows that the activation of 10 wt % W/ZSM-5 could not be achieved by thermally induced redispersion restructuring. On the other hand, we observed using operando UV–vis spectroscopy that WO_3_ was (partially) reduced before benzene production. Accordingly, H_2_ pretreatments lead to a higher degree of WO_3_ reduction and shorter activation times. However, the theory of a gradual redispersion or reduction does not explain the sudden production of CO and benzene since one would expect to monitor a steady increase of these products. This would suggest the activation of autocatalytic nature which is similar to a chemical clock reaction.^49^

## Conclusions

The activation of a zeolite W/ZSM-5 catalyst in the methane dehydroaromatization (MDA) reaction is demanding, which unfortunately results in long activation times. Through varying catalyst preparation and pretreatment parameters, and using operando and ex situ spectroscopy methods, it was found that either an optimal tungsten oxide dispersion on the zeolite surface or a reduction of the supported tungsten oxide are needed to activate the catalyst. Using this knowledge, we have proven that the activation period can be shortened or even eliminated, and that the correct pretreatment must be chosen for a certain tungsten loading range. A puzzling observation of our study is the sudden production of CO and benzene: in the case of a gradual redispersion, or reduction of the catalyst, one would expect to observe a steady increase in product formation. Our findings suggest the occurrence of an autocatalytic mechanism or chain reaction during catalyst activation, similar to a chemical clock reaction. The insights acquired from this study are of value when working toward the industrial application of MDA, as reducing the length of the activation period saves both time and energy. For such a process, one should consider using W/ZSM-5 catalysts in the medium or high weight loading regime to obtain considerable benzene yields. Furthermore, the reaction temperature also plays an important role as with lower temperatures it takes longer to activate the catalyst, but the deactivation rate is also lower, resulting in longer catalyst lifetime. In case one wants to operate at lower reaction temperatures to reduce the deactivation rate, one should contemplate whether it is economically more viable to have a catalyst prepared at higher calcination temperatures, which is active immediately, but has lower yields due to loss of acid sites. This work highlights that it is crucial to understand the catalyst’s activation mechanism in greater detail to make these decisions for an industrial relevant process.

## Data Availability

The data used in this paper has been uploaded to the YODA repository and are available under: https://doi.org/10.24416/UU01-BK6RYQ

## Supporting Information Available

ICP-OES data, physisorption data, NH_3_-TPD data, Raman spectra, calculations of the activation energy, and catalytic performance data (PDF)
