# Supplementary material for: Impact of Tungsten Loading on the Activation of Zeolite-Based Catalysts for Methane Dehydroaromatization
Source: ACS Catal. 2025 Apr 18;15(9):7241–53. doi: 10.1021/acscatal.4c07228 (PMC12053828; doi:10.1021/acscatal.4c07228)
Supplement: Supplementary file 1 — cs4c07228_si_001.pdf [file cs4c07228_si_001.pdf]

Supporting Information: The Impact of Tungsten Loading on the Activation of Zeolite-Based Catalysts for Methane Dehydroaromatization

J.J.G. Kromwijk[1], J.G.A. Vloedgraven[1], F. Neijenhuis[1], W. van der Stam[1], M. Monai[1], B.M. Weckhuysen[1,*]

*e-mail: b.m.weckhuysen@uu.nl

[1] Inorganic Chemistry and Catalysis Group, Institute for Sustainable and Circular Chemistry, Department of Chemistry, Utrecht University, 3584 CG Utrecht, Netherlands

1. Bulk Characterization

1.1 Pore volume, surface area, and chemical composition

Table S1. Pore volume and surface area of the catalyst materials under study as determined with Ar-physisorption.

Sample	Pore volume (cm^3/g)	BET (m^2/g)
ZSM-5 550 °C	0.213	444.5
2W 550 °C	0.175	335.5
2W 700 °C	0.226	433.1
5W 550 °C	0.197	401.6
5W 600 °C	0.188	388.7
5W 650 °C	0.186	377.6
5W 700 °C	0.186	381.3
7W 550 °C	0.161	335.0
7W 700 °C	0.210	407.5
10W 550 °C	0.156	283.7
10W 700 °C	0.164	302.9

Table S2. Tungsten, aluminium content, and W/Al ratios as determined with inductively coupled plasma optical emission spectroscopy (ICP-OES).

Sample	W weight loading (wt.%)	Al weight loadings (wt.%)	W/Al
ZSM-5 550 °C	-	3.41	0
2W 550 °C	1.77	3.27	0.54
2W 700 °C	1.84	3.31	0.56
5W 550 °C	4.91	3.16	1.55
5W 600 °C	5.19	3.18	1.63
5W 650 °C	5.22	3.20	1.63
5W 700 °C	5.24	3.23	1.62
7W 550 °C	6.36	3.10	2.05
7W 700 °C	6.89	3.14	2.19
10W 550 °C	9.96	2.89	3.45
10W 700 °C	10.49	3.02	3.47
10 W (E_a tests)*	9.98	-	-

* The aluminum content was not measured for this sample

1.2 Ex situ UV Vis Diffuse Reflectance Spectroscopy

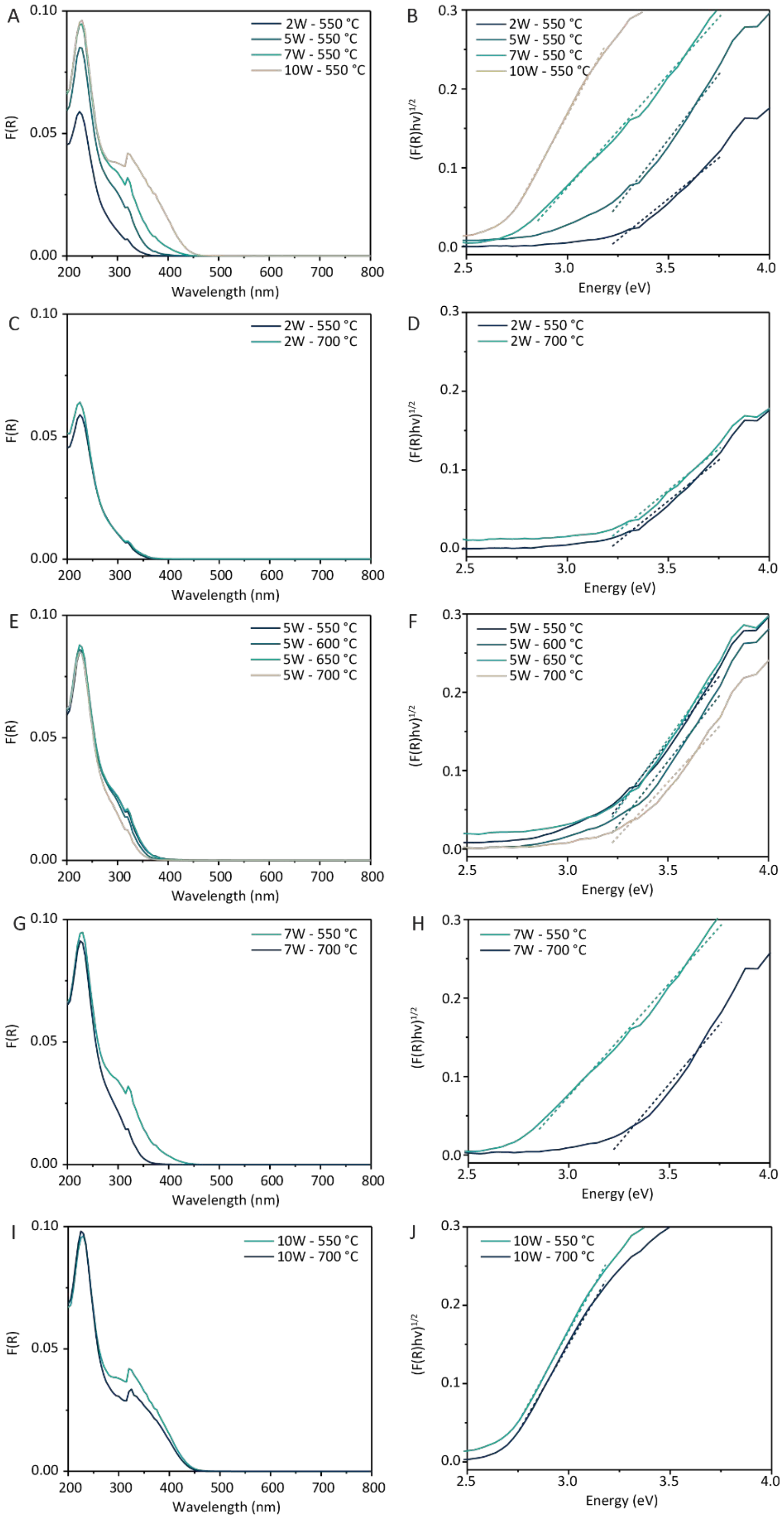

Figure S1. Ex situ UV-Vis diffuse reflectance spectra of the fresh samples and the Tauc plots for determining the edge energy (E_g) respectively of (**A,B**) the samples with varying weight loadings calcined at 550 °C, (**C,D**) 2 wt.% W/ZSM-5 calcined at 550 °C and 700 °C, (**E,F**) 5 wt.% W/ZSM-5 calcined at 550 °C, 600 °C, 650 °C, and 700 °C, (**G,H**) 7 wt.% W/ZSM-5 calcined at 550 °C and 700 °C, and (**I,J**) 10 wt.% W/ZSM-5 calcined at 550 °C and 700 °C.

In **Fig. S2** an estimation of the band gap energies obtained from the Tauc plot method are shown for the various W/ZSM-5 materials under study. Watanabe *et al.* showed that the size of the band gap is correlated with the size of WO_3 particles with bulk WO_3 having E_g values of ~2.6 eV and amorphous or nanosized WO_3 having larger E_g values of ~3.4 eV.[1] Using this method, we observe a trend; with increasing loading, the E_g value decreases, indicating larger WO_3 particles. Furthermore, we see for the medium loading regime (5 wt.% W/ZSM-5 and 7 wt.% W/ZSM-5) an increase in E_g when comparing the samples calcined at 550 °C compared to the samples calcined at 700 °C, indicating smaller WO_3 particles are present. This trend is not observed for the 2 wt.% and 10 wt.% W/ZSM-5 materials, indicating the calcination temperature does not affect the restructuring of the WO_3 in these loading regimes.

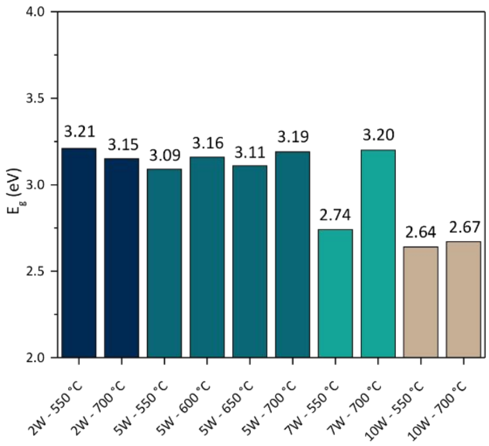

Figure S2. Band gap values obtained with the Tauc method for the various W/ZSM-5 materials under study.

1.3 Acidic properties

Table S3. Acidic properties of the catalysts materials under study as determined with NH_3-temperature programmed desorption (TPD).

	**$mmol_{NH3}/g_{cat}$**	**LT peak contribution**		**IT peak contribution**		**HT peak contribution**	
		°C	$mmol_{NH3}/g_{cat}$	°C	$mmol_{NH3}/g_{cat}$	°C	$mmol_{NH3}/g_{cat}$
ZSM-5 550 °C	1.15	242	0.59	-	-	431	0.55
ZSM-5 700 °C	0.63	211	0.32	-	-	358	0.32
2W 550 °C	0.94	230	0.49	321	0.09	413	0.36
2W 700 °C	0.77	239	0.44	353	0.12	444	0.19
5W 550 °C	0.89	221	0.44	299	0.56	386	0.38
5W 600 °C	0.81	209	0.31	279	0.22	360	0.29
5W 650 °C	0.72	212	0.42	284	0.08	376	0.22
5W 700 °C	0.63	208	0.24	289	0.22	365	0.17
7W 550°C	0.86	218	0.51	301	0.12	395	0.22
7W 700 °C	0.70	220	0.39	324	0.28	441	0.04
10W 550 °C	0.79	205	0.30	296	0.35	404	0.14
10W 700 °C	0.40	222	0.19	297	0.16	370	0.043

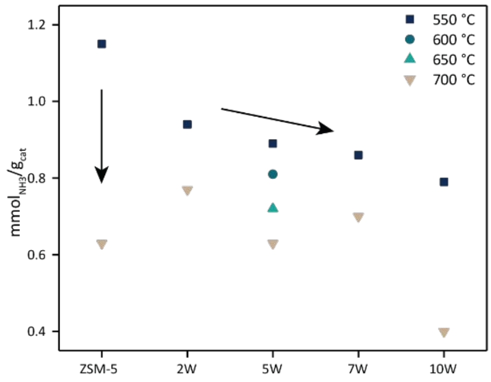

Figure S3. Trends measured with NH_3-TPD expressed as mmol ammonia adsorbed per gram catalyst as a function of tungsten weight loading and calcination temperature.

1.4 UV-Vis spectroscopy, Raman spectroscopy, and X-ray diffraction peak assignments

Table S4. UV-Vis band assignments of the hydrocarbon species and WO_{3-x} species observed in the operando experiments.

Band position (cm^{-1})	Assignment	Reference
38000-35000	Neutral aromatics	[2]
35000-30000	Charged monoenyl/cyclopentyl species	[2]
27000-25000	Charged poly-alkylated benzenes	[2]
25000-23000	Charged alkylated naphthalenes	[2]
20000-6600	WO_{3-x} localized surface plasmon resonance	[3–6]
23000-12500	Charged & neutral poly-aromatics	[2]

Table S5. Raman shifts of ZSM-5 and tungsten oxide species observed in the ex situ Raman spectra.

Peak position (cm^{-1})	Vibration	Reference
270	δ(O-W-O)	[7,8]
290	T-O-T	[9]
325	δ(O-W-O)	[7,8]
380	T-O-T	[9]
455	δ(O-Si-O(Al))	[9]
710	ν_s(O-W-O)	[7,8]
800	ν_s(O-W-O)	[7,8]

Table S6. X-ray diffraction peaks of tungsten species observed in the ex situ and in situ X-ray diffraction experiments. The reported 2Theta(°) values from the references are measured using Cu radiation.

2Theta(°)	Tungsten phase	Miller index	Reference
33.1	β-WO_3	202	[7]
33.6	β-WO_3	022	[7]
24.0	β-WO_3	220	[7]
40.4	W	110	[10]

1.5 Operando UV-Vis spectroscopy

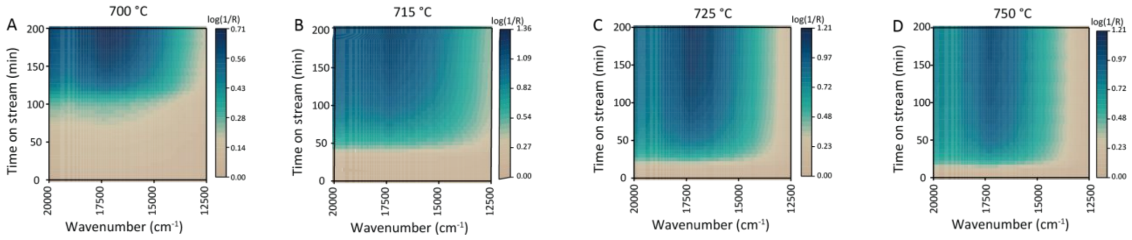

Figure S4. Contour plots of the operando UV-Vis spectroscopy measurements of the 5 wt.% W/ZSM-5 catalyst calcined at 550 °C during methane dehydroaromatization at (**A**) 700 °C (**B**) 715 °C (**C**) 725 °C (**D**) 750 °C

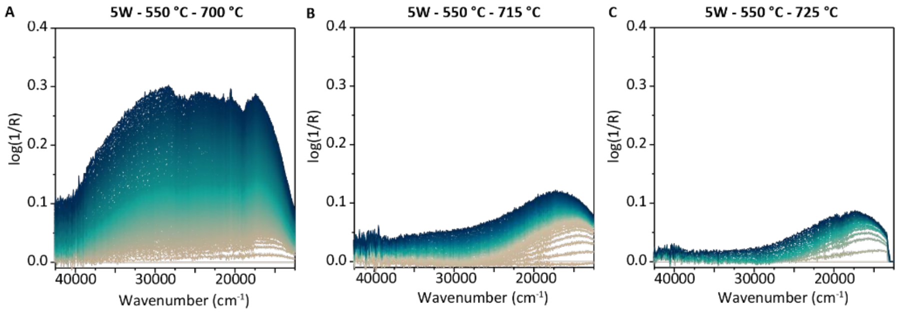

Figure S5. Operando UV-Vis spectra recorded during the activation period of the 5 wt.% W/ZSM-5 catalyst calcined at 550 °C during methane dehydroaromatization at (**A**) 700 °C (**B**) 715 °C, and (**C**) 725 °C.

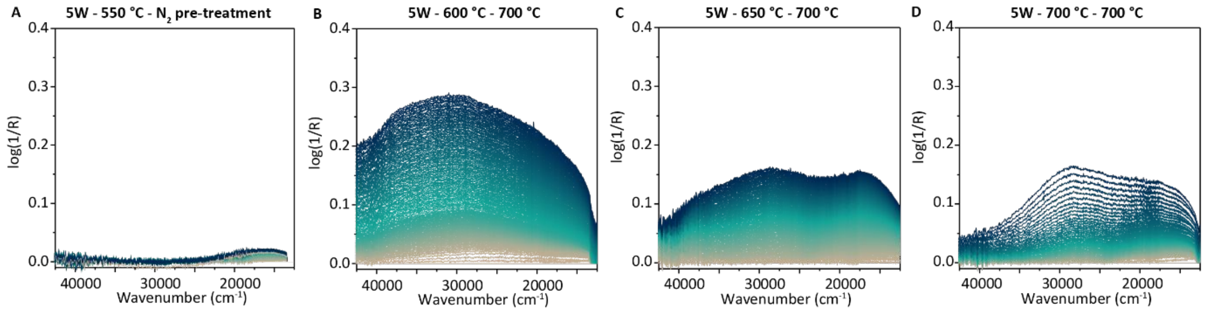

Figure S6. Operando UV-Vis spectra recorded during the activation period of the 5 wt.% W/ZSM-5 catalyst during methane dehydroaromatization at 700 °C (**A**) 5 wt.% W/ZSM-5 calcined at 550 °C, during the N_2 pretreatment (**B**) 5 wt.% W/ZSM-5 calcined at 600 °C (**C**) 5 wt.% W/ZSM-5 calcined at 650 °C, and (**D**) 5 wt.% W/ZSM-5 calcined at 700 °C.

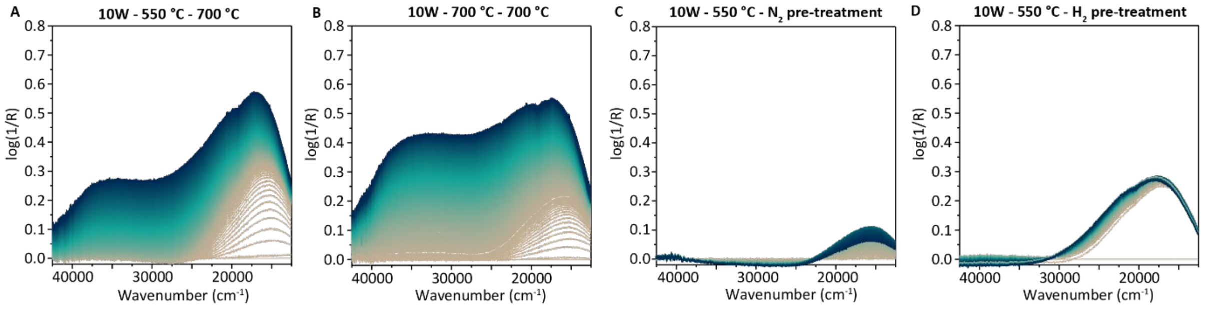

Figure S7. Operando UV-Vis spectra recorded during the activation period of the 10 wt.% W/ZSM-5 catalyst during methane dehydroaromatization at 700 °C (**A**) 10 wt.% W/ZSM-5 calcined at 550 °C (**B**) 10 wt.% W/ZSM-5 calcined at 700 °C (**C**) 10 wt.% W/ZSM-5 calcined at 550 °C during the N_2 pretreatment , and (**D**) 10 wt.% W/ZSM-5 calcined at 550 °C, during the H_2 pretreatment

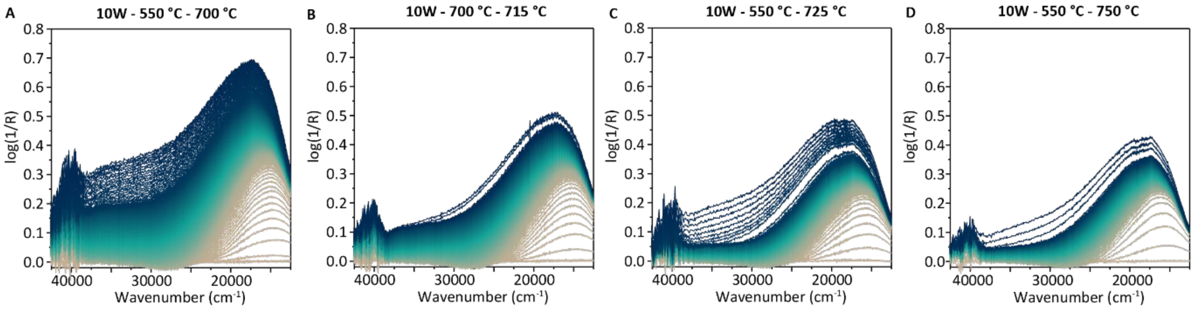

Figure S8. Operando UV-Vis spectra recorded during the activation period of the 10 wt.% W/ZSM-5 catalyst calcined at 550 °C during methane dehydroaromatization at (**A**) 700 °C (**B**) 715 °C, (**C**) 725 °C, and (**D**) 750 °C.

1.6 Raman Spectroscopy

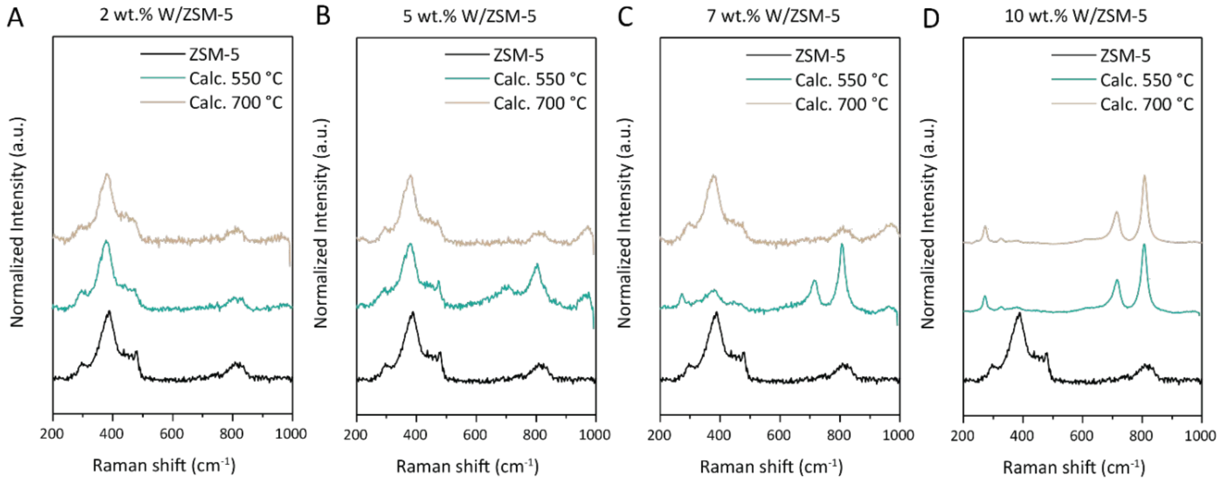

Figure S9. *Ex situ* Raman spectra of parent zeolite ZSM-5 material compared to the different catalyst materials under study: (**A**) 2 wt.% W/ZSM-5, (**B**) 5 wt.% W/ZSM-5, (**C**) 7 wt.% W/ZSM-5, and (**D**) 10 wt.% W/ZSM-5 calcined at 550 °C and 700 °C.

1.7 X-Ray Diffraction

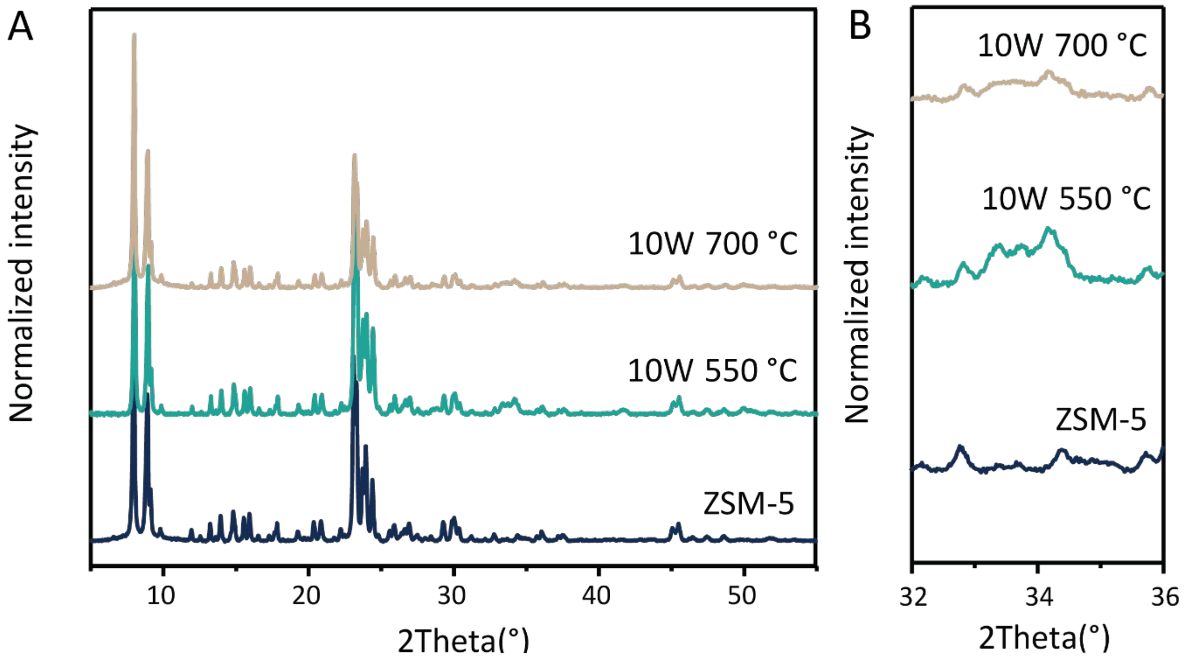

Figure S10. (**A**) X-Ray dIffraction patterns of ZSM-5 and the 10 wt.% W/ZSM-5 catalysts under study (**B**) zoom-in on the XRD pattern where the WO_3 reflections appear.

1.8 Electron Microscopy

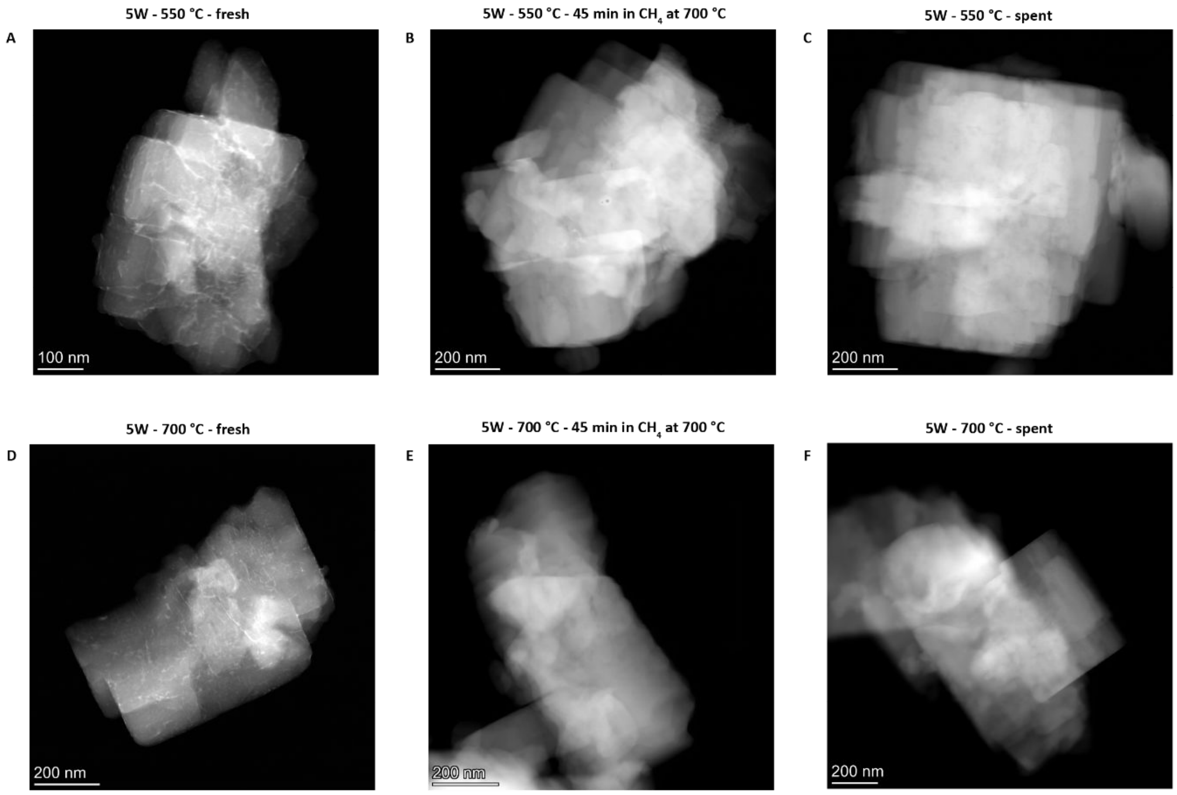

Figure S11. STEM-HAADF images of the 5 wt.% W/ZSM-5 catalyst calcined at 550 °C: (**A**) fresh, (**B**) exposed to CH_4 at 700 °C for 45 minutes, and (**C**) spent, after 10 hours of reaction. STEM-HAADF images of the 5 wt.% W/ZSM-5 catalyst calcined at 700 °C: (**D**) fresh, (**E**) exposed to CH_4 at 700 °C for 45 minutes, and (**F**) spent, after 10 hours of reaction.

2. Calculation of the activation energy

In Tables S6 and S7, the input can be found which was used to calculate the activation energy of the activation of the 5 wt.% W/ZSM-5 and 10 wt.% W/ZSM-5 catalyst materials respectively. For these calculations, the time delay before CO and benzene production of the experiments carried out at 700 °C, 715 °C, and 725 °C was used for the 5 wt.% W/ZSM-5 catalyst material. An additional temperature point at 750 °C could be used for the 10 wt.% W/ZSM-5 sample. From the time delay, the ratio of the rate constant can be calculated ($t_1/t_2 = k_2/k_1$). Using this, and assuming the pre-exponential factor, as well as the activation energy at one temperature, remains the same, the apparent activation energy could be derived. The results give very high apparent activation energies for the 5 wt.% W/ZSM-5 catalyst material with an average of 830 kJ/mol. For the 10 wt.% W/ZSM-5 sample, lower apparent activation energies with an average of 264 kJ/mol are found.

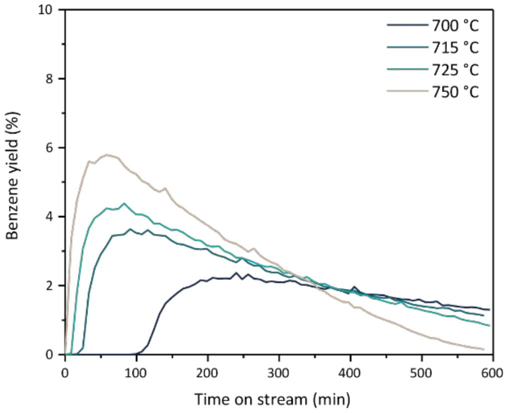

Figure S12. Benzene yields over time recorded for the 5 wt.% W/ZSM-5 catalyst at different temperatures in the 700-750 °C range. Conditions: 1 bar, 9 mL/min CH_4, and 1 mL/min N_2, 1.2 h^{-1} WHSV.

Table S7. Input used to calculate the activation energy of the activation of the 5 wt.% W/ZSM-5 catalyst material, based on the experiments carried out at 700 °C, 715 °C, and 725 °C.

T_1 (°C)	T_2 (°C)	t_1 (min)	t_2 (min)	$t_1/t_2 = k_2/k_1$	$\ln(k_2/k_1)*R$	$1/T_2-1/T_1$	R/S	E_a (kJ/mol)
700	715	100	22	4	11.5	1.56E-05	7.39E+5	739
700	725	100	8	12.5	21.0	2.57E-05	8.16E+5	816
715	725	22	8	3.125	9.47	1.01E-05	9.34E+5	934

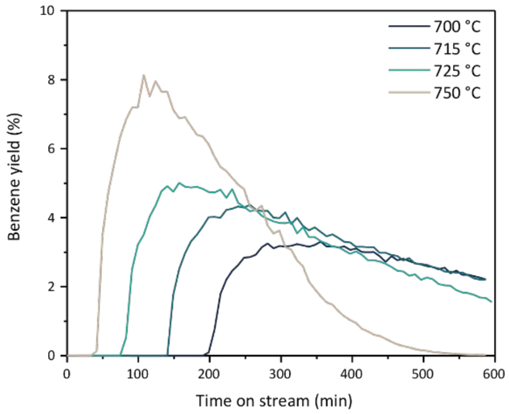

Figure S13. Benzene yields over time recorded for the 10 wt.% W/ZSM-5 catalyst at different temperatures in the 700-750 °C range. Conditions: 1 bar, 9 mL/min CH4, and 1 mL/min N2, 1.2 h^{-1} WHSV.

Table S8. Input used to calculate the activation energy of the activation of the 10 wt.% W/ZSM-5 catalyst material, based on the experiments carried out at 700 °C, 715 °C, 725 °C. and 750 °C.

T_1 (°C)	T_2 (°C)	t_1 (min)	t_2 (min)	$t_1/t_2 = k_2/k_1$	$\ln(k_2/k_1)*R$	$1/T_2-1/T_1$	R/S	E_a (kJ/mol)
700	715	215	148	1.45	3.10	1.56E-05	1.99E+05	199
700	725	215	91	2.36	7.15	2.57E-05	2.78E+05	278
700	750	215	50	4.30	12.13	5.02E-05	2.42E+05	242
715	725	148	91	1.63	4.04	1.01E-05	3.99E+05	399
715	750	148	50	2.96	9.02	3.46E-05	2.61E+05	261
725	750	91	50	1.82	4.98	2.45E-05	2.03E+05	203

3. Catalytic performance testing

3.1 Product formation

In **Fig. S14-S34** the CH_4 conversion, the yields of CO, H_2, benzene, toluene, ethane, and ethylene of all catalytic performance tests reported in this work can be found. It is to be noted that the CH_4 MFC of the used set-up leaks, causing a CH_4 spike when we switch to this gas at TOS = 0 min. Therefore, high CH_4 'conversions' are observed at the beginning of the test, which is not related to the catalytic activity. On top of that, a new 10 wt.% W/ZSM-5 sample had to be prepared for the catalytic tests at various temperatures to calculate the activation energy. Since it is challenging to exactly reproduce the catalyst from incipient wetness impregnation, the induction time slightly varies amongst these two batches. Nonetheless, the trend in induction time (10 wt.% > 5 wt.% & 7 wt.%) remains the same.

2 wt.% W/ZSM-5 – calcined at 550 °C

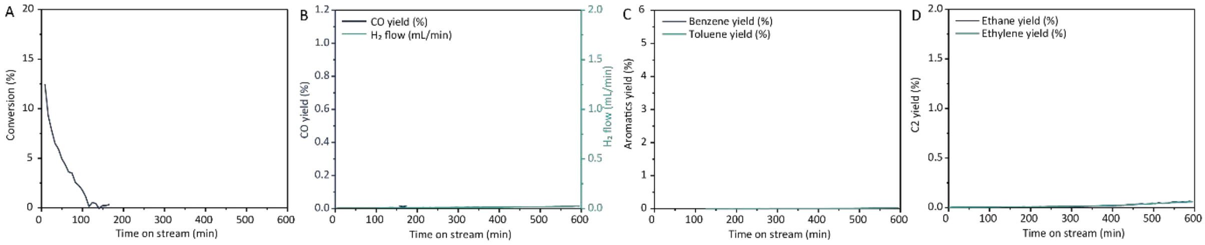

Figure S14. Catalytic performance test of the 2 wt.% W/ZSM-5 catalyst calcined at 550 °C, reaction at 700 °C in a CH_4:N_2 mixture of 9:1 mL/min. (**A**) CH_4 conversion, (**B**) CO yield and H_2 flow, (**C**) benzene and toluene yield, and (**D**) ethane and ethylene yield.

2 wt.% W/ZSM-5 – calcined at 700 °C

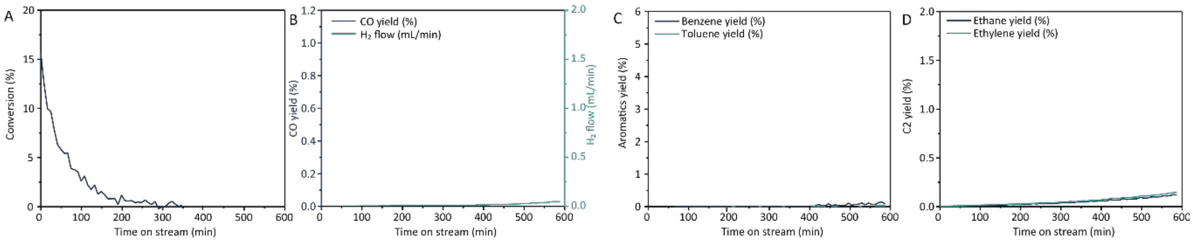

Figure S125. Catalytic performance test of the 2 wt.% W/ZSM-5 catalyst calcined at 700 °C, reaction at 700 °C in a CH_4:N_2 mixture of 9:1 mL/min. (**A**) CH_4 conversion, (**B**) CO yield and H_2 flow, (**C**) benzene and toluene yield, and (**D**) ethane and ethylene yield.

5 wt.% W/ZSM-5 – calcined at 550 °C

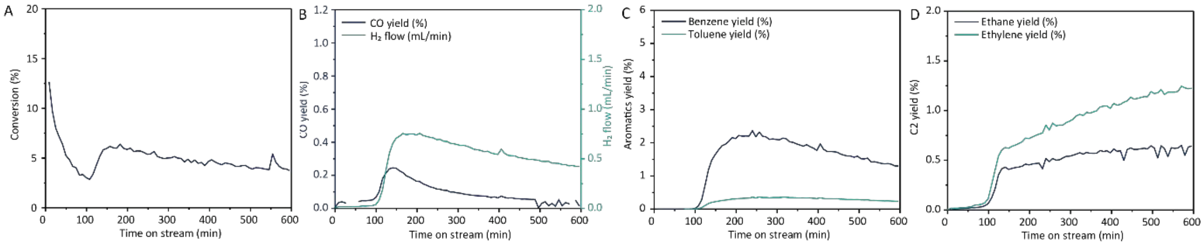

Figure S16. Catalytic performance test of the 5 wt.% W/ZSM-5 catalyst calcined at 550 °C, reaction at 700 °C in a CH_4:N_2 mixture of 9:1 mL/min. (**A**) CH_4 conversion, (**B**) CO yield and H_2 flow, (**C**) benzene and toluene yield, and (**D**) ethane and ethylene yield.

5 wt.% W/ZSM-5 – calcined at 600 °C

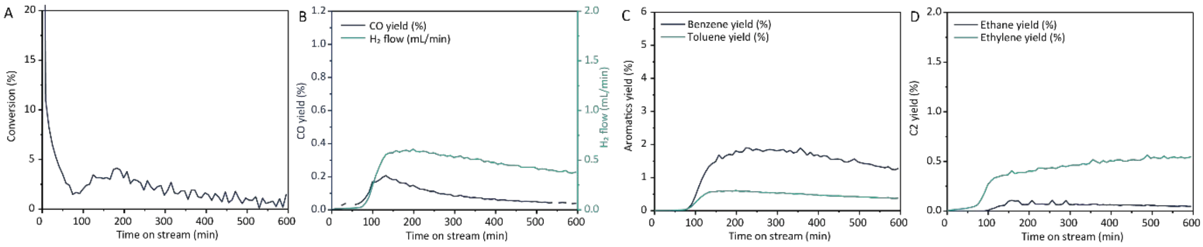

Figure S17. Catalytic performance test of the 5 wt.% W/ZSM-5 catalyst calcined at 600 °C, reaction at 700 °C in a CH_4:N_2 mixture of 9:1 mL/min. (**A**) CH_4 conversion, (**B**) CO yield and H_2 flow, (**C**) benzene and toluene yield, and (**D**) ethane and ethylene yield.

5 wt.% W/ZSM-5 – calcined at 650 °C

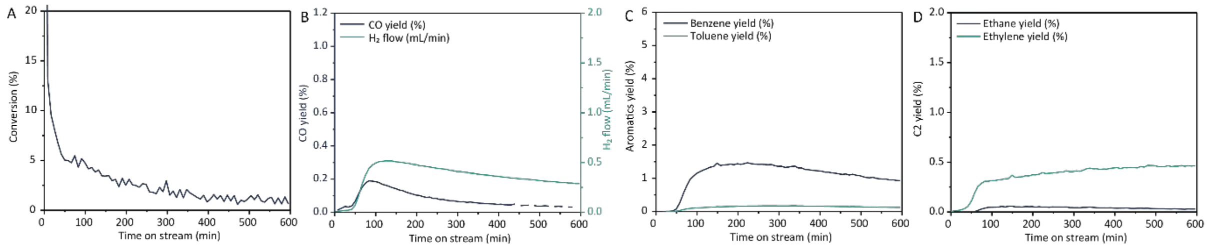

Figure S18. Catalytic performance test of the 5 wt.% W/ZSM-5 catalyst calcined at 650 °C, reaction at 700 °C in a CH_4:N_2 mixture of 9:1 mL/min. (**A**) CH_4 conversion, (**B**) CO yield and H_2 flow, (**C**) benzene and toluene yield, and (**D**) ethane and ethylene yield.

5 wt.% W/ZSM-5 – calcined at 700 °C

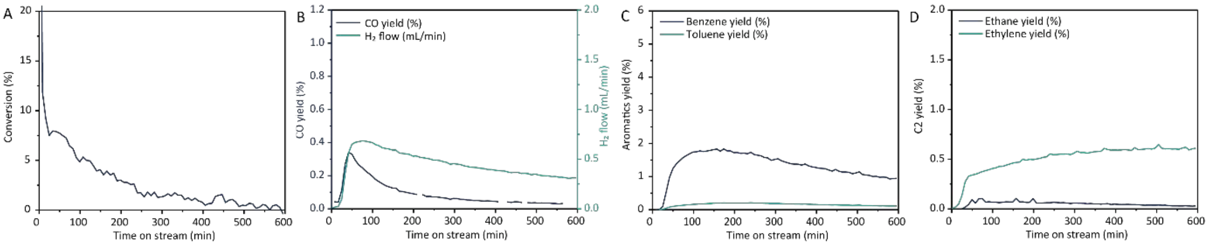

Figure S19. Catalytic performance test of the 5 wt.% W/ZSM-5 catalyst calcined at 700 °C, reaction at 700 °C in a CH_4:N_2 mixture of 9:1 mL/min. (**A**) CH_4 conversion, (**B**) CO yield and H_2 flow, (**C**) benzene and toluene yield, and (**D**) ethane and ethylene yield.

5 wt.% W/ZSM-5 – calcined at 550 °C - N_2 pretreatment

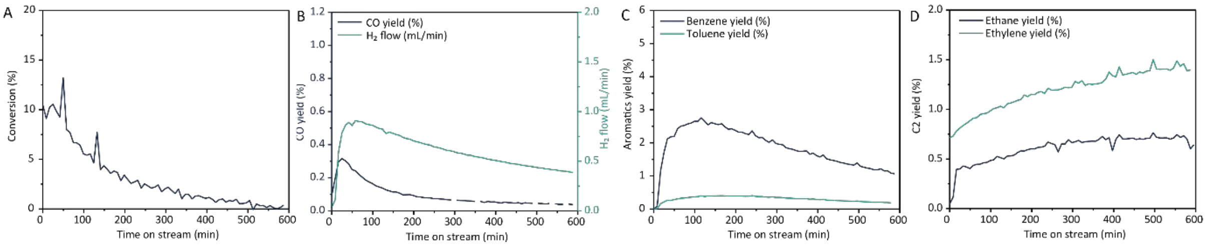

Figure S20. Catalytic performance test of the 5 wt.% W/ZSM-5 catalyst calcined at 550 °C, with a N_2 pretreatment, and reaction at 700 °C in a CH_4:N_2 mixture of 9:1 mL/min. (**A**) CH_4 conversion, (**B**) CO yield and H_2 flow, (**C**) benzene and toluene yield, and (**D**) ethane and ethylene yield.

5 wt.% W/ZSM-5 – calcined at 550 °C – reaction at 715 °C

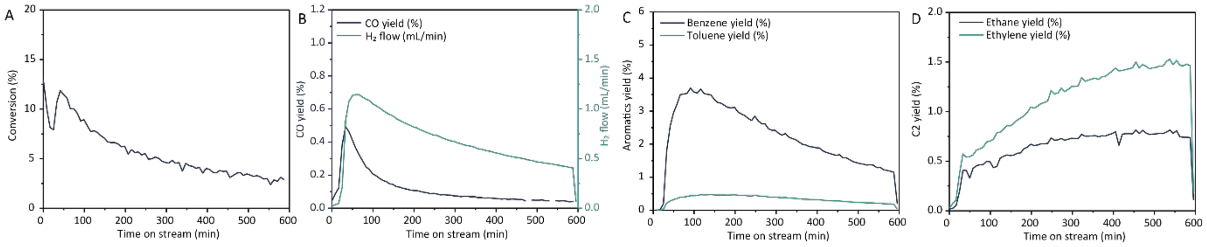

Figure S21. Catalytic performance test of the 5 wt.% W/ZSM-5 catalyst calcined at 550 °C, reaction at 715 °C in a CH_4:N_2 mixture of 9:1 mL/min. (**A**) CH_4 conversion, (**B**) CO yield and H_2 flow, (**C**) benzene and toluene yield, and (**D**) ethane and ethylene yield.

5 wt.% W/ZSM-5 – calcined at 550 °C – reaction at 725 °C

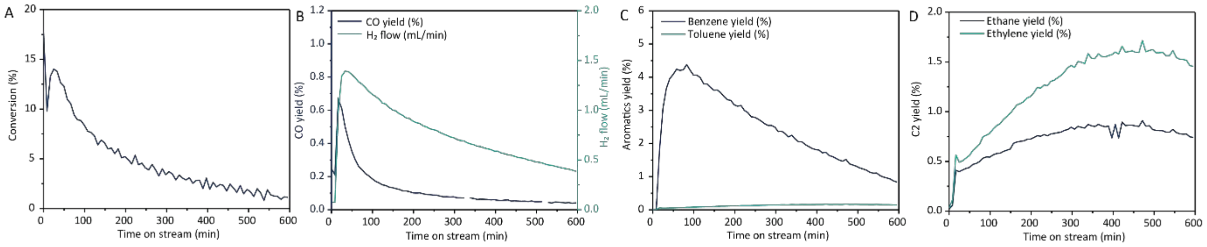

Figure S22. Catalytic performance test of the 5 wt.% W/ZSM-5 catalyst calcined at 550 °C, reaction at 725 °C in a CH_4:N_2 mixture of 9:1 mL/min. (**A**) CH_4 conversion, (**B**) CO yield and H_2 flow, (**C**) benzene and toluene yield, and (**D**) ethane and ethylene yield.

5 wt.% W/ZSM-5 – calcined at 550 °C – reaction at 750 °C

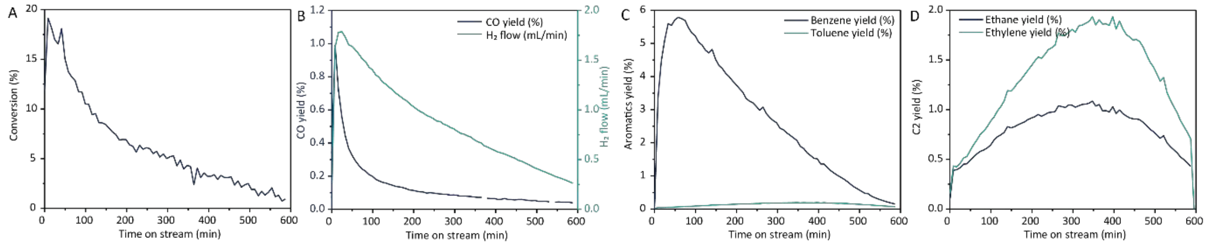

Figure S23. Catalytic performance test of the 5 wt.% W/ZSM-5 catalyst calcined at 550 °C, reaction at 750 °C in a CH_4:N_2 mixture of 9:1 mL/min. (**A**) CH_4 conversion, (**B**) CO yield and H_2 flow, (**C**) benzene and toluene yield, and (**D**) ethane and ethylene yield.

7 wt.% W/ZSM-5 – calcined at 550 °C

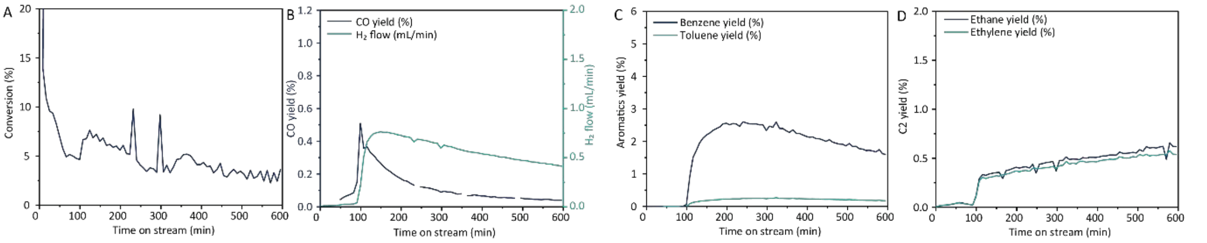

Figure S24. Catalytic performance test of the 7 wt.% W/ZSM-5 catalyst calcined at 550 °C, reaction at 700 °C in a CH_4:N_2 mixture of 9:1 mL/min. (**A**) CH_4 conversion, (**B**) CO yield and H_2 flow, (**C**) benzene and toluene yield, and (**D**) ethane and ethylene yield.

7 wt.% W/ZSM-5 – calcined at 700 °C

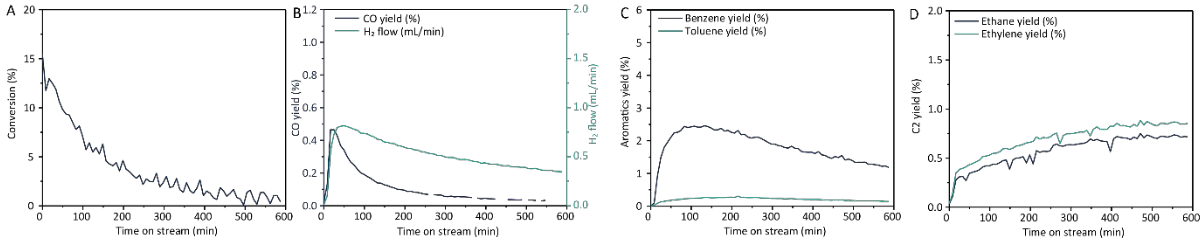

Figure S25. Catalytic performance test of the 7 wt.% W/ZSM-5 catalyst calcined at 700 °C, reaction at 700 °C in a CH_4:N_2 mixture of 9:1 mL/min. (**A**) CH_4 conversion, (**B**) CO yield and H_2 flow, (**C**) benzene and toluene yield, and (**D**) ethane and ethylene yield.

7 wt.% W/ZSM-5 – calcined at 550 °C - N_2 pretreatment

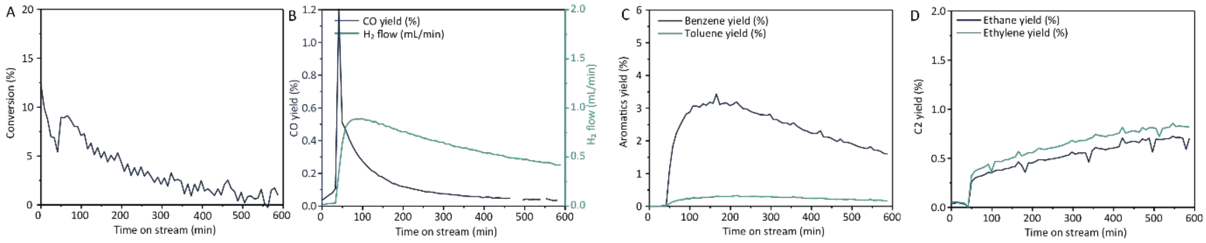

Figure S26. Catalytic performance test of the 7 wt.% W/ZSM-5 catalyst calcined at 550 °C, with a N_2 pretreatment, and reaction at 700 °C in a CH_4:N_2 mixture of 9:1 mL/min. (**A**) CH_4 conversion, (**B**) CO yield and H_2 flow, (**C**) benzene and toluene yield, and (**D**) ethane and ethylene yield.

10 wt.% W/ZSM-5 – calcined at 550 °C

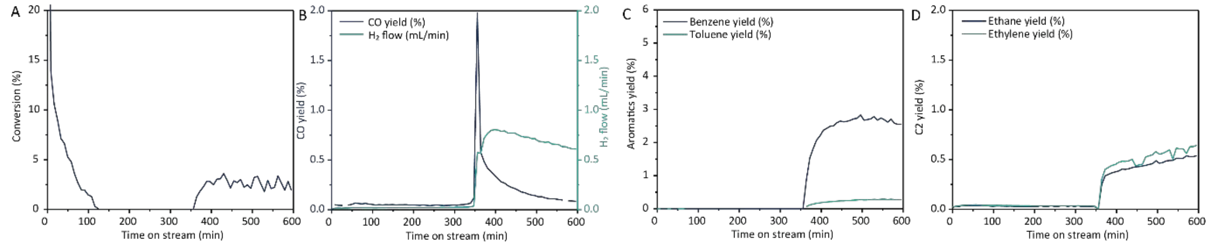

Figure S27. Catalytic performance test of the 10 wt.% W/ZSM-5 catalyst calcined at 550 °C, reaction at 700 °C in a CH_4:N_2 mixture of 9:1 mL/min. (**A**) CH_4 conversion, (**B**) CO yield and H_2 flow, (**C**) benzene and toluene yield, and (**D**) ethane and ethylene yield.

10 wt.% W/ZSM-5 – calcined at 700 °C

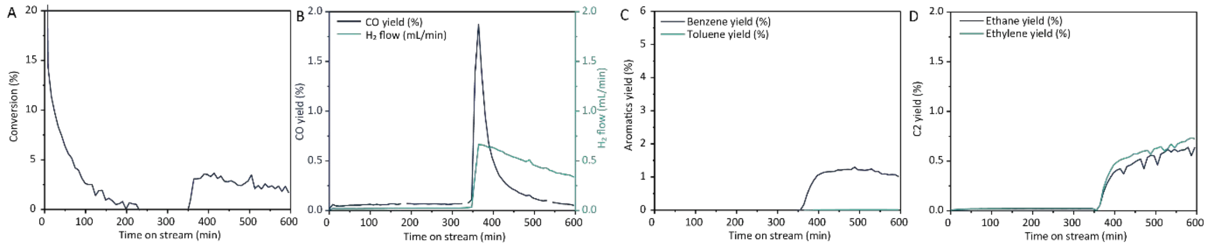

Figure S28. Catalytic performance test of the 10 wt.% W/ZSM-5 catalyst calcined at 700 °C, reaction at 700 °C in a CH_4:N_2 mixture of 9:1 mL/min. (**A**) CH_4 conversion, (**B**) CO yield and H_2 flow, (**C**) benzene and toluene yield, and (**D**) ethane and ethylene yield.

10 wt.% W/ZSM-5 – calcined at 550 °C – H_2 pretreatment

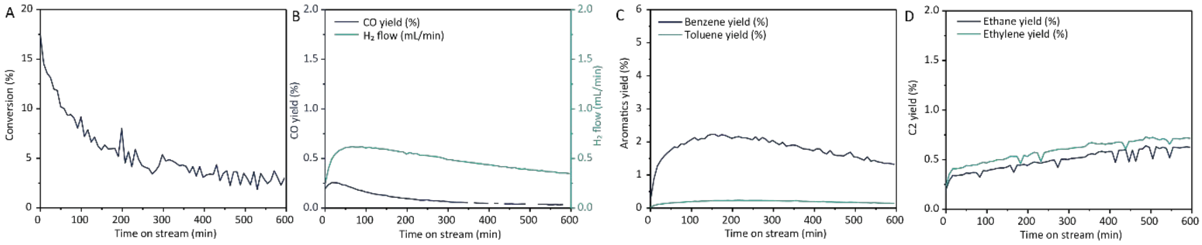

Figure S29. Catalytic performance test of the 10 wt.% W/ZSM-5 catalyst calcined at 550 °C, with a H_2 pretreatment, and reaction at 700 °C in a CH_4:N_2 mixture of 9:1 mL/min. (**A**) CH_4 conversion, (**B**) CO yield and H_2 flow, (**C**) benzene and toluene yield, and (**D**) ethane and ethylene yield.

10 wt.% W/ZSM-5 – calcined at 550 °C – N_2 pretreatment

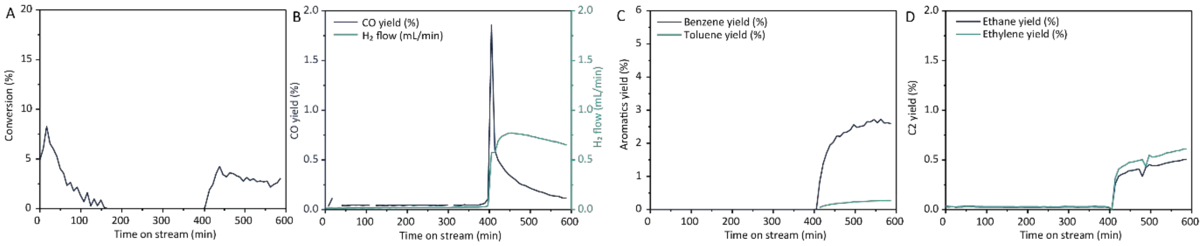

Figure S30. Catalytic performance test of the 10 wt.% W/ZSM-5 catalyst calcined at 550 °C, with a N_2 pretreatment, and reaction at 700 °C in a CH_4:N_2 mixture of 9:1 mL/min. (**A**) CH_4 conversion, (**B**) CO yield and H_2 flow, (**C**) benzene and toluene yield, and (**D**) ethane and ethylene yield.

10 wt.% W/ZSM-5 – calcined at 550 °C – 700 °C

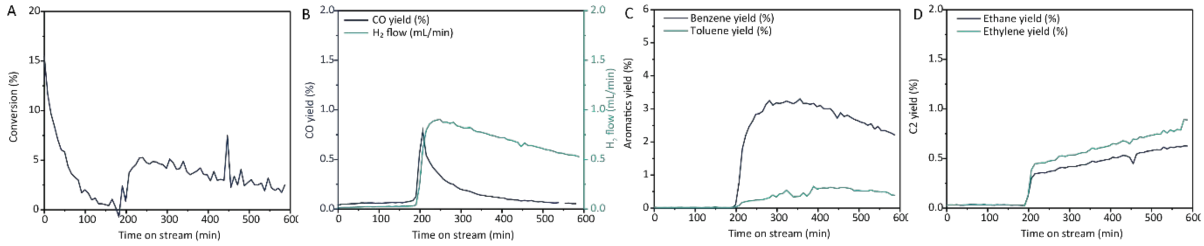

Figure S31. Catalytic performance test of the 10 wt.% W/ZSM-5 catalyst calcined at 550 °C, reaction at 700 °C in a CH_4:N_2 mixture of 9:1 mL/min. (**A**) CH_4 conversion, (**B**) CO yield and H_2 flow, (**C**) benzene and toluene yield, and (**D**) ethane and ethylene yield.

10 wt.% W/ZSM-5 – calcined at 550 °C – 715 °C

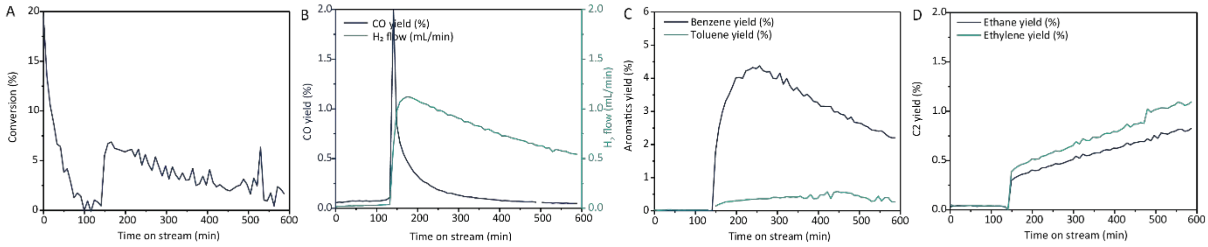

Figure S32. Catalytic performance test of the 10 wt.% W/ZSM-5 catalyst calcined at 550 °C, reaction at 715 °C in a CH_4:N_2 mixture of 9:1 mL/min. (**A**) CH_4 conversion, (**B**) CO yield and H_2 flow, (**C**) benzene and toluene yield, and (**D**) ethane and ethylene yield.

10 wt.% W/ZSM-5 – calcined at 550 °C – 725 °C

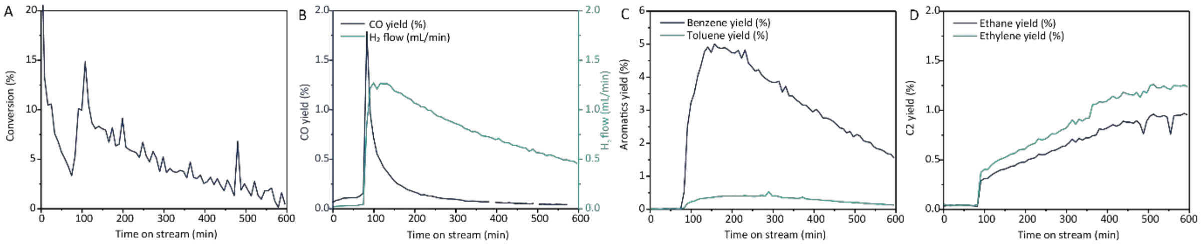

Figure S33. Catalytic performance test of the 10 wt.% W/ZSM-5 catalyst calcined at 550 °C, reaction at 725 °C in a CH_4:N_2 mixture of 9:1 mL/min. (**A**) CH_4 conversion, (**B**) CO yield and H_2 flow, (**C**) benzene and toluene yield, and (**D**) ethane and ethylene yield.

10 wt.% W/ZSM-5 – calcined at 550 °C – 750 °C

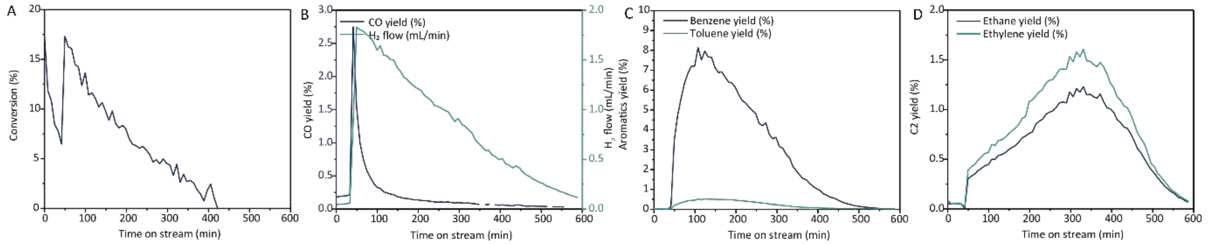

Figure S34. Catalytic performance test of the 10 wt.% W/ZSM-5 catalyst calcined at 550 °C, reaction at 750 °C in a CH_4:N_2 mixture of 9:1 mL/min. (**A**) CH_4 conversion, (**B**) CO yield and H_2 flow, (**C**) benzene and toluene yield, and (**D**) ethane and ethylene yield.

3.2 Chemical composition of spent samples

Table S9. W content of the spent samples as determined with Inductively-Coupled Plasmon Optical Emission Spectroscopy.

Sample	W weight loading (wt.%)
2W 550 °C	1.97
2W 700 °C	1.77
5W 550 °C	4.93
5W 600 °C	4.81
5W 650 °C	4.61
5W 700 °C	4.51
5W 550 °C – N_2 pretreatment	4.87
5W 550 °C – 750 °C	4.15
7W 550 °C	6.91
7W 700 °C	6.37
7W 550 °C – N_2 pretreatment	6.84
10W 550 °C	9.92
10W 700 °C	9.83
10W 550 °C – N_2 pretreatment	9.91
10W 550 °C – H_2 pretreatment	9.62

3.3 Examples of chromatograms

In Fig. S35-S38, typical chromatograms of the four different GC channels recorded during the MDA reaction can be found.

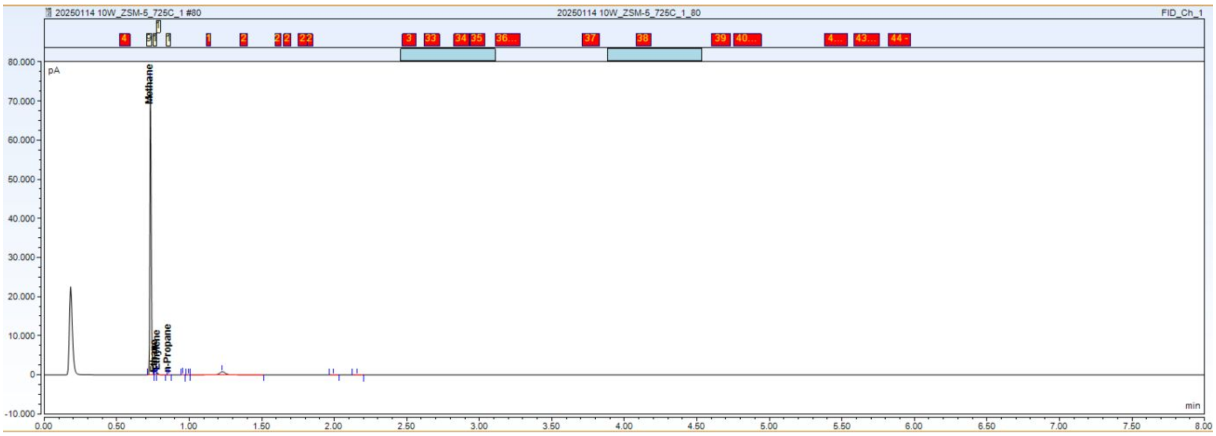

Figure S35. Typical chromatogram recorded during the MDA reaction on the first FID channel. Here, the hydrocarbons are measured.

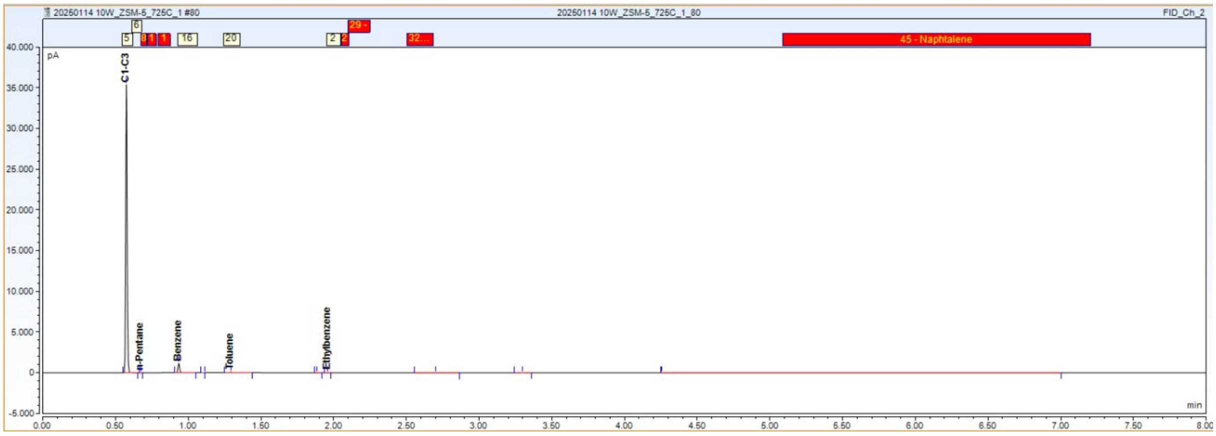

Figure S36. Typical chromatogram recorded during the MDA reaction on the second FID channel. Here, the aromatic products are measured.

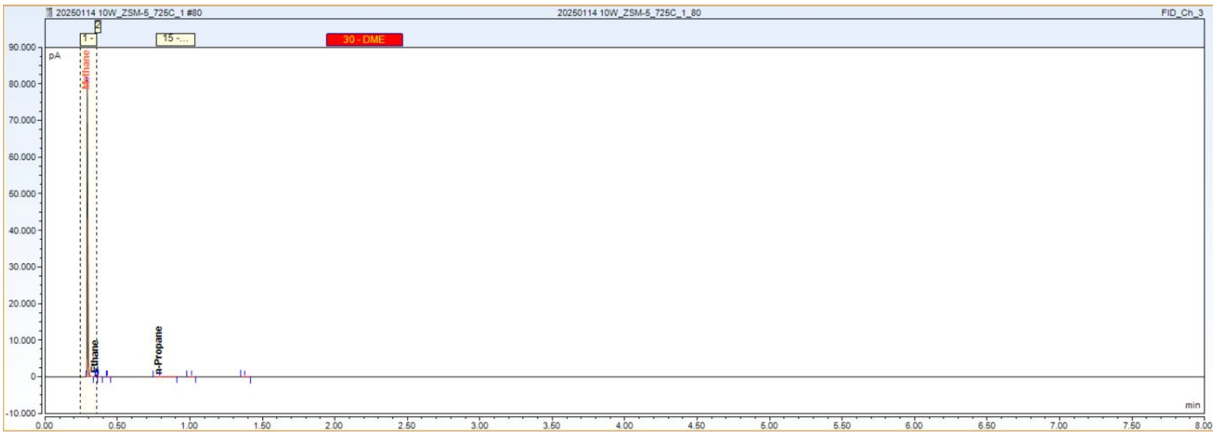

Figure S37. Typical chromatogram recorded during the MDA reaction on the third FID channel. Here, hydrocarbons are measured.

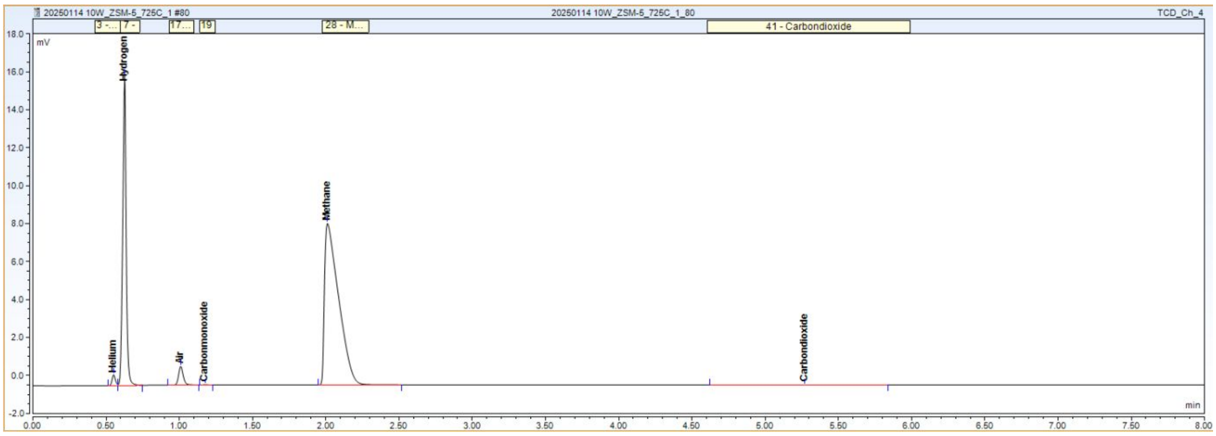

Figure S38. Typical chromatogram recorded during the MDA reaction on the TCD channel. Here, light gases (CH_4, N_2, H_2, CO,CO_2) are measured.

4. References

(1) Watanabe, H.; Fujikata, K.; Oaki, Y.; Imai, H. Band-Gap Expansion of Tungsten Oxide Quantum Dots Synthesized in Sub-Nano Porous Silica. *Chem. Commun.* **2013**, *49*, 8477–8479. https://doi.org/10.1039/C3CC44264K.

(2) Goetze, J.; Meirer, F.; Yarulina, I.; Gascon, J.; Kapteijn, F.; Ruiz-Martínez, J.; Weckhuysen, B. M. Insights into the Activity and Deactivation of the Methanol-to-Olefins Process over Different Small-Pore Zeolites As Studied with Operando UV−vis Spectroscopy. *ACS Catal.* **2017**, *7*, 4033–4046. https://doi.org/10.1021/acscatal.6b03677.

(3) Cheng, H.; Klapproth, M.; Sagaltchik, A.; Li, S.; Thomas, A. Ordered Mesoporous WO2.83 : Selective Reduction Synthesis, Exceptional Localized Surface Plasmon Resonance and Enhanced Hydrogen Evolution Reaction Activity. *J. Mater. Chem. A* **2018**, *6*, 2249–2256. https://doi.org/10.1039/c7ta09579a.

(4) Boruah, P. J.; Rakesh, ·; Khanikar, R.; Bailung, · H. Synthesis and Characterization of Oxygen Vacancy Induced Narrow Bandgap Tungsten Oxide (WO_{3-x}) Nanoparticles by Plasma Discharge in Liquid and Its Photocatalytic Activity. *Plasma Chem. Plasma Process.* **2020**, *40*, 1019–1036. https://doi.org/10.1007/s11090-020-10073-3.

(5) Manthiram, K.; Alivisatos, A. P. Tunable Localized Surface Plasmon Resonances in Tungsten Oxide Nanocrystals. *J. Am. Chem. Soc.* **2012**, *134*, 3995–3998. https://doi.org/10.1021/JA211363W.

(6) Yan, J.; Wang, T.; Wu, G.; Dai, W.; Guan, N.; Li, L.; Gong, J. Tungsten Oxide Single Crystal Nanosheets for Enhanced Multichannel Solar Light Harvesting. *Adv. Mater.* **2015**, *27*, 1580–1586. https://doi.org/10.1002/ADMA.201404792.

(7) Thummavichai, K.; Wang, N.; Rance, G.; Xia, Y.; Zhu, Y. In Situ Investigations of the Phase Change Behaviour of Tungsten Oxide Nanostructures. *R. Soc. Open Sci.* **2018**, *5*, 171932. https://doi.org/10.1098/rsos.171932.

(8) Ross-Medgaarden, E. I.; Wachs, I. E. Structural Determination of Bulk and Surface Tungsten Oxides with UV-Vis Diffuse Reflectance Spectroscopy and Raman Spectroscopy. *J. Phys. Chem. C* **2007**, *111*, 15089–15099. https://doi.org/10.1021/jp074219c.

(9) Attila, Ö.; King, H. E.; Meirer, F.; Weckhuysen, B. M. 3D Raman Spectroscopy of Large Zeolite ZSM-5 Crystals. *Chem. Eur. J.* **2019**, *25*, 7158–7167. https://doi.org/10.1002/chem.201805664.

(10) Abdallah, A. S.; Fayed, A.; Abdo, G. M.; Sallam, M. T. Phases Identifications by X-Ray Analysis for Liquid Phase Sintered Tungsten Heavy Alloys. *IJRET Int. J. Res. Eng. Technol.* **2017**, *6*, 69–79. https://doi.org/10.15623/ijret.2017.0605012.
